# Unraveling Biochemical Pathways Affected by Mitochondrial Dysfunctions Using Metabolomic Approaches

**DOI:** 10.3390/metabo4030831

**Published:** 2014-09-25

**Authors:** Stéphane Demine, Nagabushana Reddy, Patricia Renard, Martine Raes, Thierry Arnould

**Affiliations:** Laboratory of Biochemistry and Cell Biology (URBC), NARILIS (Namur Research Institute for Life Sciences), University of Namur (UNamur), 61 rue de Bruxelles, Namur 5000, Belgium; E-Mails: stephane.demine@unamur.be (S.D.); nagabushana.reddy@unamur.be (N.R.); patsy.renard@unamur.be (P.R.); martine.raes@unamur.be (M.R.)

**Keywords:** metabolites, metabolomics, mitochondria, mitochondrial uncoupling, uncoupling proteins, obesity, type II diabetes mellitus, metabolic syndrome, NMR, mass spectrometry

## Abstract

Mitochondrial dysfunction(s) (MDs) can be defined as alterations in the mitochondria, including mitochondrial uncoupling, mitochondrial depolarization, inhibition of the mitochondrial respiratory chain, mitochondrial network fragmentation, mitochondrial or nuclear DNA mutations and the mitochondrial accumulation of protein aggregates. All these MDs are known to alter the capacity of ATP production and are observed in several pathological states/diseases, including cancer, obesity, muscle and neurological disorders. The induction of MDs can also alter the secretion of several metabolites, reactive oxygen species production and modify several cell-signalling pathways to resolve the mitochondrial dysfunction or ultimately trigger cell death. Many metabolites, such as fatty acids and derived compounds, could be secreted into the blood stream by cells suffering from mitochondrial alterations. In this review, we summarize how a mitochondrial uncoupling can modify metabolites, the signalling pathways and transcription factors involved in this process. We describe how to identify the causes or consequences of mitochondrial dysfunction using metabolomics (liquid and gas chromatography associated with mass spectrometry analysis, NMR spectroscopy) in the obesity and insulin resistance thematic.

## 1. Introduction

Metabolomics can be defined as “measure of metabolite pool that exists within a cell or tissue under a particular set of environmental conditions” [1,2]. Metabolomics are often confused with metabonomics, which defines the study of metabolite modifications induced by an environmental change [3]. However, the two terms are often used interchangeably [4]. The precise definition and origins of these terms has been discussed in detail elsewhere [4]. Therefore, in this review we will use the term metabolomics to refer to both metabolomic and metabonomic studies.

Metabolomics offers an opportunity to screen and analyse several biochemical pathways at once. It also combines methods of choice for the discovery of biomarkers of a given disease. The importance of metabolomics has increased rapidly since the publication of the original article on metabonomics in 1999 [3]. Indeed, more than 7700 studies containing the word “metabolomics” have been published (results from PubMed, August 2014). Easier access to mass spectrometry facilities and decreased costs have likely led to the increase in these studies. However, we must also note that most of these studies were mainly conducted in the field of cancer biology. Since excellent recent reviews are available for this research field [5,6], cancer will not be discussed in this paper. We will rather review the metabolomic studies contributing valuable information regarding cell responses to mitochondrial dysfunction. We will first briefly review the major types of mitochondrial dysfunction(s) with a strong emphasis on mitochondrial uncoupling. We will also discuss the impact of several metabolites on uncoupling proteins’ (UCP) function and expression. We will then summarise the different metabolomic techniques/technologies that are currently available. Finally, we will review the possible implication of mitochondrial dysfunction(s) in obesity and insulin resistance and what metabolomic studies have brought to this field.

## 2. Mitochondrial Structure and Organization

Mitochondria are cellular organelles derived from an ancestor endosymbiotic α-proteobacteria that fused with an ancestor eukaryotic cell approximately 1.5 billion years ago [7]. Since this integration, mitochondria have evolved but have kept several characteristics from bacteria, such as a double cellular membrane that delimitates four different compartments: the outer mitochondrial membrane (OMM), the inner mitochondrial space (IMS), the inner mitochondrial membrane (IMM) and the mitochondrial matrix (MM). The IMM surrounds the MM, which is where major metabolic reactions take place. The IMM also forms several invaginations known as “cristaes”, which are where the mitochondrial electron transfer chain (ETC) is located. This chain is formed by four different protein complexes (I, II, III and IV); it allows the release of protons into the IMS, thus forming a proton gradient. The F_1_/F_0_-ATPsynthase (complex V) uses the gradient to catalyse ATP production.

Since these complexes have long been considered as isolated, the assembly of several complexes and other molecules, such as coenzyme Q and cytochrome C, into supercomplexes (also known as respirasomes) has been proposed [8,9]. Several supercomplexes have already been described (*i.e.*, I-III_2_-IV_1_, I-III_2_, I-III_2_-IV_2_ and I-III_2_-IV_3_-_4_) [10]. Complex I seems to be the most important complex in the assembly of supercomplexes because it is required for the assembly of any known respirasomes; however, the absence of a single complex could compromise the assembly of supercomplexes [8]. The assembly is also regulated by several proteins, including respiratory supercomplex factors 1 and 2 (Rcf1 and Rcf2), two members of the hypoxia-induced factor 1 (HIF-1) protein family [11] and human cytochrome c oxidase subunit 7A-related protein (COX7RP) [12]. In addition to the requirement of some complexes, the shape of the cristae and several lipids (such as cardiolipins and phospholipids) are also critical to the correct and stable assembly of such supercomplexes [13,14,15]. This organization into superstructures is thought to allow easier channelling of electrons through the different complexes. Interestingly, isolated supercomplexes are still able to respire by themselves [8].

Due to their key role in ATP production, mitochondria are thus usually considered the “powerhouses of the cell” [16]. However, recent decades have seen the discovery of many new functions and participation by mitochondria in the regulation of several crucial events, such as cell death [17], mitophagy [18], calcium homeostasis [19,20] and cell signalling [16,21]. Because these functions are beyond the scope of this review, we will only focus on the role of mitochondria in metabolism and its handling of metabolites.

## 3. Mitochondria and Metabolism

**Figure 1:**
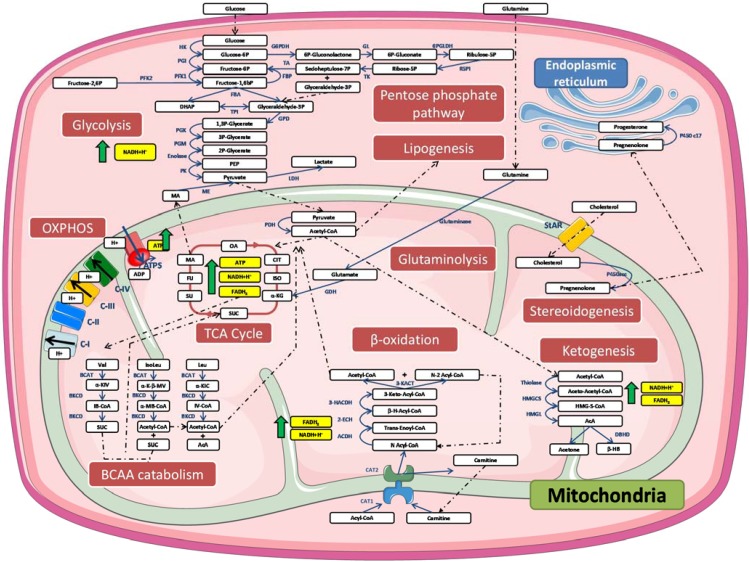
Simplified representation of biochemical pathways related to mitochondria.

Glucose (through glycolysis), free fatty acids (FFAs) and glutamine (through glutaminolysis) are major fuels in mammalian cells [22]. Several enzymatic reactions are vital to the complete oxidation of these molecules in mitochondria. Therefore, mitochondria have always been considered important organelles in the regulation of metabolism. Indeed, many processes occur at least partially in the mitochondria, such as the β-oxidation of free fatty acids, ketogenesis, glutaminolysis, branched amino acid (BCAA) catabolism and interested readers will find a description of major enzymatic reactions in the tricarboxylic acid cycle (TCA, also known as the Krebs cycle or citric acid cycle) and especially important for cardiomyocytes [23]. In this section, we will discuss all these different but interconnected metabolic pathways. Figure 1 provides a description of the major enzymatic reactions.

One of the most described functions of mitochondria is the β-oxidation of FFAs (also occurring in peroxisomes for long fatty acyl chains); this function is especially important for cardiomyocytes [24]. This process begins with the conversion of FFAs into acyl-CoA esters by the acyl-CoA synthase, which is a cytosolic process. After this conversion, the acyl-CoA ester is transported into the mitochondria. To allow the translocation of acyl chains containing more than 14 carbons [25], the acyl-CoA is transferred into the mitochondria by the carnitine acyl-carnitine translocase system [25]. Once imported into the mitochondrial matrix, the different steps of the β-oxidation process take place: dehydrogenation, hydratation, second step of dehydrogenation, thiolysis and production of a shorter acyl-CoA (removal of two carbons).

Acetyl-CoA could also come from glycolysis. Indeed, cells can also metabolize glucose in the cytosol in order to produce pyruvate through several enzymatic reactions (detailed in Figure 1). In anaerobic conditions, this metabolite will be converted into lactate by the lactate dehydrogenase. However, in aerobic conditions, pyruvate will be imported into the mitochondria where it will be converted into acetyl-CoA by the pyruvate dehydrogenase. It must also be noted that glycolysis does not only serve to fuel the TCA cycle and to produce ATP. Indeed, it has been proposed that glycolysis could also provide “building blocks” for the synthesis of other metabolites. For instance, glucose-6-phosphate and fructose-6-phosphate, two intermediates from glucose catabolism, can be used to synthesize nucleotides such as uridine/guanosine/cytidine 5’-mono-, di-, and triphosphates. Another intermediate, glyceraldehyde-3-phosphate could be used for the synthesis of complex lipids such as phospholipids, sphingolipids, cerebroside or gangliosides. Finally, 3-phosphoglycerate can be used for the synthesis of amino acids such as serine, glycine or cysteine. These pathways have been reviewed in details elsewhere [26].

The acetyl-CoA molecules formed will then be used to feed other metabolic pathways, such as the TCA cycle [26], or to produce cholesterol or other lipids through lipogenesis [27]. The acyl-CoA can also be transported back to the cytosol by the carnitine acyl-carnitine translocase system to produce acetoacetate and β-hydroxybutyrate (ketone bodies) in ketogenic tissues [3]. These enzymatic reactions are detailed in Figure 1. During starvation, the peripheral tissues will be able to import these ketone bodies into mitochondria once released into the blood stream, where they will be degraded into acetyl-CoA, which will eventually be used to replenish the TCA cycle. The ketone bodies can also be used to aliment lipogenesis and cholesterol synthesis, especially in the brain, lactating mammary glands and the liver [28]. The biological relevance of the non-oxidative metabolism of ketone bodies has been demonstrated recently both *in vivo* and *in vitro*. Indeed, the knock-down of AACS leads to the impaired differentiation of murine 3T3-L1 adipocytes and lower cholesterol blood concentration in mice [29]. All these enzymatic pathways have been discussed in a recent review [28].

As mentioned before, the acetyl-CoA can finally be completely oxidized in the TCA cycle. All the enzymes involved in this cycle are localized into the mitochondrial matrix except the succinate dehydrogenase, which is located in the IMM and is part of the mitochondrial complex II. During the TCA cycle, NADH and FADH_2_ will be produced. The close proximity between the TCA cycle and the oxidative phosphorylation system allows the rapid use of three and one molecule(s) per cycle of formed NADH and FADH_2_, respectively, to produce ATP.

Glutaminolysis is another of the most recently identified metabolic pathways that occur in mitochondria. The degradation of glutamine, which is a very abundant amino acid, is used to produce energy. This process is particularly important in both non-cancerous and cancerous cells because the privation of glutamine completely prevents cell growth [29]. Glutaminolysis begins in humans with the uptake of glutamine by the plasma membrane antiporter solute carrier family 7 member 5 (SCL7A5) [30]. Some cells (*i.e.*, hepatocytes [31]) expressing the glutamine synthetase can also directly synthesize glutamine from glutamate and ammonia, thus providing the substrate for glutaminolysis. Once imported into the cell, glutamine can be used in the cytosol to produce new purine nucleotides [32,33] or glucosamine-6P (through the activation of glucosamine-6P synthase [34]); glutamine can also be used for protein synthesis. However, glutamine can also be imported and degraded into glutamate and ammonia in the mitochondria by glutaminase [35]. The deamination of glutamate leads to the formation of α-ketoglutarate and therefore feeds the TCA cycle to produce energy. Alpha-ketoglutarate can also be reduced in acetyl-CoA by isocitrate dehydrogenase-1 to aliment lipogenesis [36]. Although this pathway seems functional in any models, cells grown under hypoxia seem to rely exclusively on this process for lipogenesis [36].

In addition to the degradation of glutamine, mitochondria are also involved in the metabolism of three other hydrophobic amino acids: valine, leucine and isoleucine (also known as branched amino acids [BCAAs]). Even if every tissue is virtually able to catabolize BCAAs, this activity is more important in some tissues (especially skeletal muscle and adipose tissues) when compared with the kidneys or intestines [37,38]. BCAA degradation is performed by two enzymes: (1) branched-chain amino acid aminotransferase (BCAT) isoenzyme [39] and (2) branched α-keto acid dehydrogenase (BCKD). Two BCAT isoforms resulting from the expression of two different genes comprise the mitochondrial and cytosolic pools [39]. However, the mitochondrial enzyme seems to be predominant as a cytoplasmic activity and is only found in the brain [39]. This leads to the production of acyl-CoA from these intermediates. Ultimately, the formed acyl-CoA can be oxidized in the mitochondria to produce energy. The understanding of BCAA metabolism’s importance has increased in recent years [40]. BCAAs are able to regulate the activity of peroxisome proliferator-activated receptor γ (PPARγ) [41], which is a key transcription factor in mitochondrial biogenesis [42] and adipocyte differentiation [43]. They also regulate the expression of 8-oxyoguanine DNA glycosilase 1, which is a protein involved in oxidative stress-induced DNA damage repair [44]. In addition, BCAAs also play a key role in regulating macroautophagy because they can activate the mechanistic target of rapamycin (mTOR), thereby inhibiting this process [41,45]. BCAAs also play an important role in glucose metabolism because exposure to BCAAs increases glucose uptake in rat skeletal muscles [46,47] and murine adipocytes [48], which suggests a positive role for these amino acids on insulin sensitivity.

Finally, mitochondria are also involved in synthesising steroids; this process is known as steroidogenesis. This process requires cholesterol, which can have different origins: (1) synthesized *de novo* in endoplasmic reticulum; (2) imported through the uptake of high-density lipoprotein (HDL); or (3) imported through the uptake of low-density lipoprotein (LDL); or (4) extracted from lipid droplets [49]. To allow the synthesis of steroids, cholesterol is translocated into mitochondria by the steroidogenic acute regulatory protein (SaRT) transporter family [49]. Once imported, cholesterol is converted to a pregnenolone cytochrome P450-linked side chain cleaving enzyme (P450scc) and exported back to the cytosol and the endoplasmic reticulum, where the final steps of steroidogenesis will be performed [49].

The metabolic pathways are regulated by substrate concentrations, intermediary metabolites or the final products produced during different enzymatic reactions. Metabolites can regulate the activity of metabolic enzymes in different manners: allosteric regulation, enzyme abundance (by regulating its degradation or transcription) and post-translational modifications in enzymes such as phosphorylation, sumoylation and acetylation. Many papers have demonstrated acetylation’s control of several enzymes’ activity. For instance, the acetylation of phosphoenolpyruvate carboxykinase 1 (PEPCK1) [50,51], an enzyme fulfilling one key step of gluconeogenesis (Figure 1), increases its interaction with the ubiquitin protein ligase E3 component n-recognin 5 (UBR5). This interaction leads to the ubiquitylation of PEPCK1 and its degradation by the proteasome. Interestingly, the acetylation of proteins requires the substrate NAD^+^. Therefore, virtually any conditions leading to a decreased NAD^+^/NADH ratio could severely interfere with the availability of NAD^+^ and the acetylation of (mito)proteins, which could lead to dramatic changes in enzymatic pathway activity. These mechanisms have been reviewed [52].

Glucose represents the main source of energy in mammalian cells. Glucose is catabolized through a process known as glycolysis to form NADH and pyruvate. Pyruvate is imported into mitochondria, where it is converted into acetyl-CoA that must enter the TCA cycle to be completely oxidized. This leads to the formation of NADH and FADH_2_. Another important source of energy is the amino acid glutamine, which is very abundant. To produce energy, glutamine can be used through a process called glutaminolysis. Other amino acids can be used for ATP production, such as branched-chain amino acids. Another important source of energy in some cell types, such as cardiomyocytes, or in cases involving glucose deprivation is free fatty acids. Their degradation occurs during a process known as β-oxidation, which occurs in the mitochondrial matrix. Finally, NADH and FADH_2_ are used by the mitochondrial OXPHOS to produce ATP. During electron transit in the complexes, protons accumulate in the IMM. The F_1_/F_0_-ATPsynthase uses this proton gradient to produce energy as ATP. Another biochemical process partially occurs in mitochondria: steroidogenesis. Abbreviations: 2-ENCH: 2-enoyl-CoA hydratase; 3-HACDH: 3-hydroxylacyl-CoA dehydrogenase; 3-KACT: 3-ketoacyl-CoA thiolase; ACDH: acyl-CoA dehydrogenase; ATPS: mitochondrial ATPsynthase; BCAT: branched-chain aminotransferase; BKCD: branched-chain alpha-ketoacid dehydrogenase; C I: mitochondrial complex I; C II: mitochondrial complex II; C III: mitochondrial complex IIII; C IV: mitochondrial complex IV; CAT1: carnitine acyltransferase 1; CAT2: carnitine acyltransferase 2; DBHD: d-β-hydroxybutyrate dehydrogenase; FBA: fructose bisphosphate aldolase; G6PDH: glycerol-6-phosphate dehydrogenase; 6PGLDH: 6-phosphogluconate dehydrogenase; GDH: glutamate dehydrogenase; GL: gluconolactonase; GPD: glyceraldehyde phosphate dehydrogenase; HK-1: hexokinase 1; HMGCS: HMG-CoA synthase; HMGL: HMG-CoA lyase; LDH: lactate dehydrogenase; ME: malic enzyme; OXPHOS: oxidative phosphorylation chain; P450c17: cytochrome P450c17; P450scc: P450 side-chain cleavage enzyme; PDH: pyruvate dehydrogenase; PFK-1: phosphofructokinase 1; PFK-2: phosphofructokinase 2; PGK: phosphoglycerate kinase; PGI: phosphoglucose isomerase; PGM: phosphoglycerate mutase; PK: pyruvate kinase; R5PI: ribose-5-phosphate isomerase; StAR: steroidogenic acute regulatory proteins; TA: transaldolase; TCA cycle: tricarboxylic cycle; TK: transketolase; TPI: triose phosphate isomerase; α-K-B-MV: α-keto-b-methylvarate; α-KIC: α-ketoisocaproate; α-KIV: α-ketoisovalerate; α-KG: α-keto-glutarate; α-MB-CoA: α-methylbutyryl-coenzyme A; β-H-Acyl-CoA: β-hydroxy-acyl-coenzyme A; β-HB: β-hydroxybutyrate; AcA: acetoacetate; CIT: citrate; DHAP: dihydroxyacetone phosphate; FADH_2_: flavin adenine dinucleotide; FU: fumarate; H^+^: proton; HMG-S-CoA: 3-hydroxy-3-methyl-glutaryl-CoA; IB-CoA: isobutyryl-coenzyme A; ISO: isocitrate; IsoLeu: isoleucine; IV-CoA: isovaleryl-coenzyme A; Leu: leucine; MA: malate; OA: oxaloacetate; NADH: nicotinamide adenine dinucleotide; PEP: phosphoenolpyruvate; SUC: succinate-coenzyme A; Suc-CoA: succynyl-coenzyme A; SU: succinate; Val: valine.

## 4. Mitochondrial Dysfunction

### 4.1. Different Types of Mitochondrial Dysfunction

Because mitochondria play key roles in several metabolic pathways, a dysfunction (that could have many origins) in these organelles will have severe consequences on the biology of the cells. However, mitochondrial dysfunctions are not only represented by defects in metabolic pathways. Mitochondrial dysfunctions are classically defined as impairments in numerous mitochondrial function indexes including OXPHOS, respiration, membrane potential, proton leak and ROS (reactive oxygen species) production [53]. These mitochondrial dysfunctions could be caused by many cellular conditions, such as mitochondrial and genomic DNA mutation(s) [54,55], supercomplex destabilization [56], endoplasmic reticulum stress [57] and mitochondrial protein aggregates [58]. In this review, we will only discuss mitochondrial uncoupling and its consequences on metabolism and cellular functions.

### 4.2. Mitochondrial Uncoupling and UCP Protein Family

Mitochondrial uncoupling can be defined as the dissipation of the IMM proton gradient, which leads to heat production and a decrease in ATP synthesis by the mitochondrial ATPsynthase [59]. This process is often considered to be a mitochondrial dysfunction. However, a mild mitochondrial uncoupling can be activated with certain physiological conditions and can contribute to non-shivering adaptive thermogenesis by brown adipose tissue (in rodents, humans and animals that hibernate) [60]. Mitochondrial uncoupling is mainly mediated by UCPs (a family of IMM transporters) and adenine nucleotide translocases (ANTs).

In humans, four isoforms of ANT are found; they present tissue specificity. The ANT1 is located in skeletal muscle, the heart and the brain [61]. ANT2 is essentially expressed in non-proliferative cells [62]. ANT4 is only found in testis [63] while ANT3 is ubiquitously expressed [62]. ANTs play a role in regulating mitochondrial uncoupling [64] by controlling the ADP/ATP ratio. Although its affinity for ADP is low, it compensates with a high protein abundance in mitochondria, which allows the induction of a mild mitochondrial uncoupling. The structures and functions of ANTs have been reviewed elsewhere [65].

In mammals, the UCP family contains five different members [66]. The initial function proposed for these proteins was the transport of protons from the IMS to the mitochondrial matrix [67]. This process dissipates the proton gradient established between the IMS and the mitochondrial matrix, inhibits ATP production with F_1_/F_0_ ATPsynthase (a process known as mitochondrial uncoupling) and produces heat instead of ATP [68]. The five proteins present different tissue localization. UCP-1 is mostly expressed in brown [69] and brown-in-white (brite) adipocytes [70]. UCP-2 is broadly expressed but is found abundantly in the spleen, the kidneys (in Kupfer cells [71]), immune cells, cells from the central nervous system, the pancreas, the heart and brown adipose tissue [72,73,74,75]. Its expression is also higher in human head, neck, skin, prostate and pancreatic tumours [76]. The third member, UCP-3, is abundantly but not exclusively expressed in skeletal muscles [71] and it has been found in pancreatic islet beta cells [77]. UCP-4 and UCP-5, which is also known as brain mitochondrial carrier protein-1 (BMCP1), are predominantly expressed in the brain [78] and liver; UCP-5 is predominately expressed in skeletal muscle [79]. Even though UCPs have been intensively investigated since their initial discovery in 1985 [67,80], the functions of the other members are still highly debated [68,81] despite the clear implication of UCP-1 in thermogenesis through the activation of mitochondrial uncoupling [60,67,80]. The different functions proposed for each UCP are detailed in Table 1.

**Table 1:** Summary of localization and functions proposed for the different UCPs.

	Localization	Function(s) proposed	Species	Tissue or cell-type	Reference
**UCP-1**	Brown adipocytes, Beige adipocytes, Skeletal muscle	ROS production limitation	Mouse	Skeletal muscle cells	[82]
		Mitochondrial uncoupling	Mouse	Brown adipose tissue	[83]
		Thermogenesis	Mouse	White adipose tissue	[84]
**UCP-2**	Spleen, Kupfer cells, Immune cells, Central nervous system, Pancreas, Heart, Brown adipocytes	Mitochondrial uncoupling	Yeast	/	[74]
		ROS production limitation	Mouse	RAW264.7 macrophages	[85]
			Mouse	Endothelial cells	[86]
			Human	Pancreatic adenocarcinoma cell lines	[87]
		Malate, oxaloacetate and aspartate transporter	Human	HepG2 hepatocarcinoma cells	[88]
**UCP-3**	Skeletal muscle, Pancreatic islet beta cells	Mitochondrial uncoupling	Mouse	Skeletal muscle	[89]
		Fatty acid anion transport (hypothetical)	/	/	[90]
		Ca^2+^ ion transport	Human	EA.hy926 endothelial cells	[91]
**UCP-4**	Central nervous system	Limited proton translocation	/	Artificial liposomes	[92]
		Chloride ion transport	/	Artificial liposomes	[92]
		Mitochondrial uncoupling with increased ROS production	Mouse	3T3-L1 white adipocytes	[93]
		Limitation of ROS production	Human	SH-SY5Y neuroblastoma cells	[94]
		Regulation of mitochondrial complex II activity	Human	SH-SY5Y neuroblastoma cells	[94]
			C. Elegans	/	[95]
**UCP-5**	Central nervous system, Liver, Skeletal muscle	Limited proton translocation	/	Artificial liposomes	[92]
		Chloride ion transport	/	Artificial liposomes	[92]
		Limitation of ROS production	Human	SH-SY5Y neuroblastoma cells	[94]
		Regulation of mitochondrial complex II activity	Human	SH-SY5Y neuroblastoma cells	[94]

In addition to the regulation of mitochondrial uncoupling, two crucial new functions have been proposed recently for UCPs. The first is the regulation of ROS production by limiting the rate of the mitochondrial respiration ETC. However, UCPs are not the only way to regulate ROS production; cells have many enzymes designed for this function, such as the superoxide dismutase (SOD) enzyme family members. Three SODs have been described: SOD1, SOD2 and SOD3. Even though their function is similar (they catalyse the conversion of two molecules of superoxide to dioxygen and hydrogen peroxide), these enzymes differ in their mechanisms and localizations. SOD1, also known as Cu/Zn SOD, and SOD3 use Cu and Zn ions in their catalytic centres. They are expressed in cytoplasmic and extracellular compartments, respectively. SOD2 is localized in the mitochondrial matrix and uses Mn ions to degrade superoxide molecules. Regulation and functions in diseases involving SODs has recently been reviewed elsewhere [96]. The hydrogen peroxide formed by SODs can then be degraded in water by different enzymes from the catalase, thioredoxin (Trx) and peroxiredoxin families. The first is essentially localized in peroxysomes and fulfils its function because of the heme ferriprotoporphyrin IX, which is localized in its active site [97]. Three isoforms are found for thioredoxins in humans: TrxR1 (expressed in cytosol); TrxR2 (restricted to mitochondria); and thioredoxin glutathione reductase (TGR), which is specific to testis [98]. The peroxiredoxin family members (Prxs) detoxify several ROS such as peroxide, peroxynitrite and organic hydroperoxides. The family contains six enzymes that can be subdivided into three groups: 1-Cys (Prx6), typical (Prx1-4) and atypical 2-Cys Prxs (Prx5). Peroxiredoxins show different localization: Prx3 is restricted to mitochondria, while the other Prxs are found in the cytosol, nucleus, Golgi apparatus and peroxisomes. The functions and regulations of Prxs have been discussed in detail elsewhere [99]. The last family of ROS detoxifying enzymes is the glutaredoxins (Gxrs). These enzymes play a pivotal role in reducing protein disulphide bonds by glutathione by transferring two electrons on two glutathione molecules and producing a glutathione disulphide that can be reduced in glutathione by glutathione reductase. The four isoforms found in humans possess different localizations. Grx2a is mainly mitochondrial [100].

UCPs’ role in the transport of metabolites, such as chloride and fatty acid anions, has also been reported. Uncoupling of the mitochondrial electron transfer chain by UCPs could also decrease the ATP/ADP ratio and thereby modify the abundance of several metabolites such as NAD^+^, which is a metabolite required for the deacetylation of many mitochondrial mitochondrial proteins by Sirtuin 3 [101]; affecting deacetylation affects the activity of many mitochondrial proteins such as metabolic enzymes [52]. UCPs could thus play a role in regulating several metabolites. In the next part of this review, we will discuss how UCPs could interfere with metabolic pathways/metabolites and the putative effect of several metabolites on mitochondrial uncoupling.

## 5. Mitochondrial Uncoupling Proteins and Metabolites Crosstalk

### 5.1. Glucose

Glucose can modulate the expression of UCP family members both *in vitro* and *in vivo*. In primary cultures of bovine pericytes and retinal capillary endothelial cells maintained in cultures during four media passages with different glucose concentrations (5, 23 and 30 mM), ROS production increased according to the glucose concentration and the expression of UCP-1, UCP-2 and SOD2 [102]. One possible explanation for this is the attempt to decrease ROS production through the induction of mitochondrial uncoupling. However, this compensatory mechanism was not observed in glucose concentrations above 30 mM, thus suggesting a threshold-dependent ROS regulation [102]. Similar results were obtained with human umbilical vein endothelial cells [103]. EAhy926 endothelial cells in cultures with high glucose concentrations also respond with an increased cellular Q10 amount; increased ROS abundance; and higher activity in several glycolytic enzymes, such as hexokinase I, lactate dehydrogenase and acyl-CoA dehydrogenase, which is associated with increased expression of UCP-2 and SOD2 [104]. Even if still not completely elucidated, the increase in UCP-2 expression in glucose-rich conditions could rely on the activation of insulin signalling mediated by Akt activation and FoxO1 phosphorylation, which is a post-translational modification that allows the sequestration of the transcription factor from the nucleus [105]. A link between the FoxO1 mutation (FLAG-tagged UCP-2Δ256) and increased UCP-2 expression has also been demonstrated *in vivo* in FVB-BL6 hybrid mice [106].

### 5.2. Nucleotides

Both UCP-1 and ANT can be inhibited by purine nucleotides such as GDP [107]. The binding of GDP to UCP-1 is dependent on pH and is optimal at pH 7.0. In acidic conditions (pH 4.0), GDP binding is less important because of the presence of the carboxyl group at E190, which is situated in the fourth transmembrane helix of UCP-1 [108]. The carboxyl group impairs nucleotide binding to the hydrophobic binding pocket found in the third domain of UCP-1, which would accommodate the binding of purine nucleotides [109]. The protonation of two other amino acids situated on the cytosolic side of the protein could also determine access to the nucleotide binding site [110].

### 5.3. Amino Acids

Evidence of the regulation of mitochondrial uncoupling by some amino acids has emerged in recent years. Valine, leucine or isoleucine deprivation in C57BL/6J mice is sufficient to shut down lipogenesis and trigger lipolysis through UCP-1 expression in brown adipose tissue [111,112]. This suggests that the expression of UCP-1 can be regulated by the availability of some amino acids. These effects could be because of the activation of the sympathic nervous system and the release of corticotrophin-releasing hormone (CSH) by the hypothalamus after the activation of the stimulatory G protein/cAMP/protein kinase A/cAMP response element-binding protein (CREB) pathway, as demonstrated in C57BL/6J mice [113].

### 5.4. Lipids

Several lipid classes have regulatory roles on UCP expression and mitochondrial uncoupling regulation. First, FFAs seem to play an important role in this regulation [114]. Some authors have demonstrated that FFAs possess a protonophoric effect, depending on their chain length. Long chain FFAs (between C12 and C16) and long unsaturated FFAs with sizes over half of the mitochondrial membrane thickness, seem to have the most potent effect [114]. The effect of FFAs on the mitochondrial electron transport chain has been investigated recently in isolated mitochondria from rat hearts and livers [115]. This study revealed that FFAs can significantly decrease the electron transport in complexes I and III. This is accompanied by a decrease in mitochondrial membrane fluidity and an increase in ROS production. However, FFAs seem to have the opposite effect in cases involving the reverse electron transport chain [115], thus suggesting that FFAs’ effect on mitochondria might depend on the direction of the electron flow. In the hearts of rats treated with HFD (high fat diet), a condition that induces intracellular accumulation of FFAs, increases can be seen in fatty acid β-oxidation, mitochondrial thioesterase 1 activity, and UCP-3 protein associated with mitochondrial uncoupling [116]. As demonstrated in isolated rat muscle cells, this effect might rely on superoxide anion production, NFκB activation and iNOS induction (NO production) [117]. However, the role of UCP-3 as a functional uncoupling protein is still debated and controversial [107]. Another mechanism by which FFAs could control mitochondrial uncoupling in brown adipocytes is through a direct effect on UCP-1. FFAs, such as palmitate, can physically interact with UCP-1, thus leading to a conformational change in the protein and its accelerated enzymatic proteolysis [118]. This study shows that FFAs therefore bind and regulate UCP-1 in competition with nucleotides. Other results suggest that UCP-1 can also act as an H^+^/long chain fatty acid symporter, at least in isolated brown adipose tissue in mice [119]. Interestingly, “non-flippable” fatty acids (such as glucose-O-ω-laurate (12 C), glucose-O-ω-palmitate (16 C), glucose-O-ω-stearate (18 C) and glucose-O-ω-arachidate (20 C) cannot activate UCP-1, thus suggesting that fatty acid orientation is important in the activation of UCP-1 [115]. In addition to the direct control of UCP-1 activity, lipids can also modulate UCP-1 expression. The administration of all-trans-retinoic acid, beta-carotene omega-dicarboxylic acid, which is a non-metabolizable fatty acid analogue, can trigger UCP-1 induction [115]. Medium-chain FFAs seem to be the most potent UCP-1 activators compared to other FFAs [116]. This effect might rely on superoxide anion production, NFκB activation and iNOS induction (NO production), as demonstrated in isolated rat muscle cells [117]. However, it should be mentioned that the role of UCP-3 as a functional uncoupling protein is still debated and controversial [107].

Interestingly, polyunsaturated fatty acids (PUFAs) are even more efficient than saturated fatty acids in the up-regulation of UCP1 expression [120]. Treating obese Wistar rats with docosahexaenoic acid leads to improvements in mitochondrial function and decreased mitochondrial uncoupling [121]. This can be explained by docohexaenoic acid’s positive effect on PPARγ activity, which is a transcription factor that regulates the expression of UCPs [122]. Another group has also reported increased UCP-1 expression in brown adipocytes in mice that were fed a PUFA-rich diet [123]. However, to prevent docohexaenoic acid’s side effects in Wistar rats (such as diastolic dysfunction [124]) while keeping the beneficial effect of obesity, administering an eicosapentaenoic acid/docosahexaenoic acid 1:1 ratio to rats is recommended [121]. The positive effect of eicosapentaenoic acid on mitochondrial uncoupling is due to either an up-regulation of UCP-2 expression through the stimulation of eNOS (and NO release) mediated by AMPK activation [125] or an increased mitochondrial biogenesis (through the activation of proliferator-activated receptor-gamma coactivator-1α [PGC-1α] and nuclear respiratory factor 1 [Nrf1]) as demonstrated in the epididymal adipose tissue of C57BL/6J-treated mice [126]. This increases mitochondrial biogenesis and increases the beta-oxidation rate for FFAs [126] while reducing the free fatty acid level and triglyceride content in this adipose tissue. Several studies have also confirmed the beneficial effect of PUFAs. Janoska *et al.* [127] demonstrated that the administration of long-chain n-3 polyunsaturated fatty acids could limit fat deposits and stimulate fatty acid re-esterification in epididymal adipose tissue in mice exposed to high fat diet, even if no evidence of a role for UCP-1 was found in these conditions. This effect is thus most likely not dependent on cold-induced thermogenesis. These effects have been attributed to decreases in the synthesis of pro-inflammatory lipid mediators, such as 15-deoxy-Δ (12,15)-prostaglandin J (2) and protectin D1 [128]. In this study, the increase in mitochondrial oxidative capacity was observed, and no sign of mitochondrial uncoupling was found. The beneficial effects of PUFAs on the limitation of hyper-adiposity and some associated symptoms of metabolic syndrome might be mainly independent of mitochondrial uncoupling. Their positive effect would rather be because of their activating effect on PPARα, PPARδ and PPARγ [122,129].

Lipoic acid, which is a compound derived from octanoid acid, also presents interesting features. This metabolite can inhibit the mitochondrial electron transport chain and ATP production [130], thus triggering the activation of PGC-1α and leading to increased expression of UCP-2 and several genes participating in FFA β-oxidation and lipogenesis [131,132]. Lipoic-acid-treated rats display and increase the abundance of glucose transpoter 4 (GLUT4) protein at the plasma membrane of muscle cells and therefore reduced glucose concentrations in the blood stream [133], a process that has been confirmed in 3T3-L1 adipocytes [134]. These combined effects might explain lipoic acid’s beneficial effect on insulin sensitivity [131,135].

Other lipid-derived metabolites play important roles in controlling mitochondrial activity. For example, 3-hydroxydecanoic, 3-hydroxydodecanoic, 3-hydroxytetradecanoic and 3-hydroxypalmitic acids are metabolites known to accumulate in the blood of patients suffering from long-chain 3-hydroxyacyl-CoA dehydrogenase deficiency. These different molecules seem to act as potent mitochondrial uncouplers, thus leading to decreases in the mitochondrial potential membrane, reductions in matrix NADPH and a lower ATP/O ratio (which reflects the amount of ATP produced from a single oxygen molecule) accompanied by decreased H_2_O_2_ production [136]. Mitochondrial coupling is also regulated by lipid metabolites, including arachidonic acid-derived molecules such as epoxyeicosatrienoic acids (EETs). The inhibition of EET production by a selective epoxygenase inhibitor (MS-PPOH) in neonatal hippocampal astrocytes induces a strong reduction in the mitochondrial potential membrane, fragmentation of the mitochondrial network and lower oxygen consumption by the organelle, which could be reverted with EET supplementation [137].

The peroxidation of lipids leading to the formation and accumulation of several metabolites, including 4-hydroxy-2-nonenal (HNE), is a well-known hallmark of obesity, especially in tissues displaying low-grade inflammation, such as visceral adipose tissue. HNE activates mitochondrial uncoupling through the expression and activation of several uncoupling proteins [138] or through direct increases in proton conductance of the IMM [139].

Electrophilic, nitrated lipids could also play an important role in modulating mitochondrial uncoupling. In a study performed on the perfused hearts of C57/BL6 mice, nitro-linoleate and nitro-oleate can bind and modify ANT1 through nitroalkylation on Cys57 [140]. This modification seems to confer cardioprotection against ischemia-reperfusion by a mechanism that depends on the activation of mitochondrial uncoupling [140].

We have seen so far that some particular classes of lipids could lead, under certain circumstances, to mitochondrial dysfunction and mitochondrial uncoupling largely mediated by the modulation of the activity and/or expression of UCP proteins and ANT1. However, the expression of some UCPs can also have an effect *per se* on lipid metabolism. Evidence supporting this hypothesis has been demonstrated for UCP-3. In UCP-3^−/−^ mice receiving a high-fat diet (HFD) treatment, the phosphorylation of Akt (Ser473) and AMPK (Thr172) was reduced and accompanied by a lower abundance of GLUT4 [141]. These mice also presented signs of increased insulin resistance and decreased fatty acid oxidation. Interestingly, wild-type mice exposed to an HFD show similar effects, thus suggesting that an HFD *per se* can down-regulate the expression of UCP-3 and GLUT 4 and can reduce Akt and AMPK activation [141].

### 5.5. Ketone Bodies

The putative impact of ketone bodies on UCP expression is demonstrated by the fact that C57BL/6J mice exposed to a ketone-enriched diet seemed to over-express UCP-1 in brown adipose tissue [142]. Exposed to a similar diet, the brains of Wistar rats responded with increased expression of both UCP-4 and UCP-5 [143]. Similar increases in UCP-2, UCP-4 and UCP-5 were also seen in the hippocampi of juvenile C3HeB/Fe mice exposed to a ketogenic diet [144]. In this case, the increase in UCP expression is sufficient to significantly decrease maximum mitochondrial respiration rates and ROS production of isolated mitochondria from these cells [144]. However, an opposite relation between ketone body synthesis and UCP-1 expression has been found in female mice sterilized with ovariectomies [145], thus suggesting that the relationship between ketone body production and UCP-1 is not observed in every model.

If ketone bodies seem to have a regulatory effect on some UCP expression, some UCPs also have a direct effect on ketone body production. The overexpression of UCP-1 increases ketone body synthesis in human stromal fibroblasts [146]. NMRI mice treated for four days with retinoic acid, which increases the expression of UCP-1 [147] and UCP-2 and limits fat deposits in liver [148], show increases in circulating ketone bodies [147,148], thus confirming the link between ketone bodies and UCP expression regulation. However, in contrast to UCP-1 and UCP-2, the overexpression of UCP-3 in C57/BL6J mice livers (by transduction with lentivirus coding for UCP-3) does not alter the concentration of β-hydroxybutyrate [149], which is a ketone body, thus suggesting that some UCPs are not involved in regulating ketone body synthesis.

### 5.6. Effect of Plant Metabolites on Mitochondria

Dietary consumption of some metabolites can also control mitochondrial uncoupling and ROS production. Flavonoids are a large group of secondary plant metabolites that have been described as potent antioxidant compounds [150]. However, it is now commonly accepted that some flavonoids seem to be more efficient than others. Indeed, quercetin and galangin are far more efficient than taxifolin and catechin in preventing mitochondrial membrane lipid peroxidation induced by Fe^(2+)^/citrate because quercetin can scavenge 2,2-diphenyl-1-picrylhydrazyl and superoxide radicals [151]. Other plant metabolites such as fucoxanthin, which is a carotenoid present in some edible brown seaweeds, can induce UCP-1 over-expression in white adipose tissue and subsequently activate mitochondrial uncoupling [150]. Moreover, it confers a strong anti-obesity effect by improving insulin resistance and the normalization of glucose concentrations in the blood stream; it also possesses a potent antioxidant effect [152]. Fucoxanthin could therefore be of interest in the treatment of obesity and cancer and in promoting healthier ageing.

We have seen that many metabolites can affect the expression of the UCP family. The opposite is also true: the alteration of UCP expression also modifies the concentrations of many metabolites. However, limited data are available for some classes of metabolites, such as ketone bodies or food complements. This suggests the critical importance of screening metabolites in studies where an effect on UCPs is observed to obtain a better understanding of their regulation. In the next part of this review, we will discuss the different metabolomic techniques that can be used for this purpose.

## 6. Metabolomic Techniquest

Several metabolites are able to strongly effect mitochondrial uncoupling induction and the mitochondrial function. Techniques allowing the simultaneous identification and quantitative analysis of several metabolites have been very helpful in better understanding how metabolites affect mitochondrial (dys)function and the effect of mitochondria activity and their alterations on metabolite signatures. The term metabolomics refers to the study of several metabolites at a given time under certain conditions [3]. While this set of techniques is built on other high-throughput analytical levels, it offers a serious advantage over the other “-omics” techniques such as transcriptomics, proteomics or genomics. Metabolite concentrations are amplified compared to the extent of the expression of the corresponding gene and provide a more integrative level of addressing the functionality of metabolic network maps [153,154]. Therefore, metabolomics are very sensitive compared to other techniques and provide a dynamic, functional integration of metabolism [155]. We can identify four major categories of metabolomics studies [156]. The first type of study is referred to as “metabolite profiling”, in which authors attempt to identify every change in a metabolite in their experimental model without any pre-defined candidates in mind [156]. However, the possibility of detecting and quantifying all metabolites modified in a given experimental condition is a challenge because of the low amount of some molecules and their instability. In addition, the metabolites to be analysed can vary greatly (qualitatively and quantitatively) in time and spatial organization (referring to the highly dynamic aspect of metabolomes). Furthermore, metabolomes are not only influenced by the disease and/or treatment (condition of interest to study) but also by the method used for sample preparation. Finally, the metabolites represent a numerous and complex group of molecules showing a high amount of diversity; they are also very heterogeneous in reactivity and physico-chemical properties. According to Beecher *et al.* [157], more than 5,000 metabolites are of interest in humans. If we also consider secondary metabolites, this number is even higher [158]. Some authors have reported more than 200,000 different metabolites in plants [159]. In addition, the very different concentrations of these metabolites can pose challenges in their detection by mass spectrometry. In the second category of metabolomic studies (or “targeted metabolomics”), the strategy is to only focus on a limited number of molecules that have been pre-selected as candidates of interest to reduce the complexity and the time required for analysis [156]. The third category of metabolomic studies is “metabolite fingerprinting”. In this approach, authors do not attempt to identify the metabolites but rather define a “fingerprint” of the metabolites available for a particular time-point and condition of interest. Because this technique requires less time for analysis and computations, it is the technique of choice in the discovery of “biomarkers” and diagnostics [156]. Another powerful approach is the quantitative metabolomic. In this type of study, an extra step is needed to label the metabolites of interest (with radioactive or stable isotopes). One of the most-used protocols involves using ^13^C-labelled glucose. Other metabolites can also be labelled using this stable isotope. By using labelled metabolites, the secondary-derived metabolites’ abundance can be tracked and quantified using standard metabolomic approaches. However, the necessity of using a labelling procedure could be particularly challenging in human studies; therefore, this technique is mainly used in fundamental research performed on laboratory animals or cell models [160]. Once the metabolites have been labelled, mass spectrometry is used to determine the concentrations of metabolites of interest in different samples.

**Figure 2:**
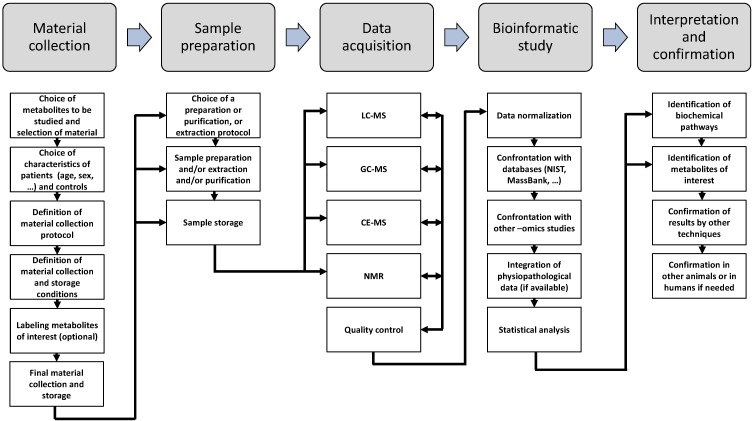
Schematic representation of workflow usually used in metabolomics study.

A typical workflow in a metabolomic study includes several experimental phases: sample collection; metabolite extraction or isolation; and final analysis of metabolites using nuclear magnetic resonance (NMR) [161] and liquid [162] or gas chromatography [163] coupled with mass spectrometry (LC-MS and GC-MS, respectively). The spectra generated by mass spectrometry must be analysed and compared to databases [164] to identify the metabolites present in the samples [1]. A typical metabolomic workflow is presented in Figure 2. In the next part of this review, we review the principles and interests of the main metabolomic techniques available.

A metabolomics study is usually divided into five parts: material collection, sample preparation, data acquisition, bioinformatic analysis and interpretation of data obtained. Before collecting the material of interest, several steps must be followed. First, a suitable material must be chosen based on the metabolites to be studied. Second, the animal model or the characteristics of the humans used during the study must be considered. Suitable controls must also be defined at this point. Third, the material collection protocol and storage conditions must be adapted to ensure the preservation of metabolites of interest. Radiolabelling is usually performed at this step if required. In the second step, the material is prepared to allow the extraction of the metabolites of interest or to allow the material to be used with the instruments in the next step. In the third step, the metabolites of interest are detected in the samples using different techniques, including LC-MS, GC-MS, capillary electrophoreses coupled with mass spectrometry (CA-MS) or nuclear magnetic resonance (NMR). Ideally, a quality protocol step is added to the protocol to ensure the proper detection of metabolites. Once the spectra are obtained, they must be compared to databases (either free of charge or commercial) to identify the metabolites detected. Ideally, any information regarding the physio-pathological state of the patients (if applicable) and data from other “-omics” studies must be added to the database created. Statistical analysis is then performed to isolate metabolites that are differentially abundant in the samples and identify pathways modified in a particular condition. Finally, the modifications found must be confirmed in other animal models and/or humans to ensure their biological relevance.

### 6.1. NMR Spectroscopy

One way to analyse the metabolites in a particular biological specimen is by using NMR. This allows simultaneous identification and quantification of a wide range of metabolites. This technique presents several advantages. First, it requires little or no preparation of the biological material, which can be useful in avoiding bias due to the isolation of metabolites of interest. Second, this simplicity also allows the automation of this step [156]. Third, the technique is reasonably rapid compared to other mass spectrometry techniques, and many samples (200–300 samples) can be analysed in one day [156]. Fourth, this technique is non-destructive; therefore, the same sample can be used for different purposes [156], which is useful in studies conducted with valuable human material. Fifth, the technique is reproducible, including between different departments or institutions [156]. Sixth, the technique could be sensitive depending on equipment used (up to the ng level, according to the instrument considered) [156]. Several kinds of NMR exist. The most-used type is simple pulse sequence and one-dimensional nuclear Overhauser enhancement spectroscopy (NOESY) with water suppression [155]. If the sample complexity is high, the 2D NMR technique is recommended to reduce the complexity of the sample [155]. NMR is also compatible with ^13^C labelling of metabolites even though this requires longer acquisition times. Stable isotope-resolved metabolomics have been reviewed elsewhere [160]. Labelling metabolites is also possible in isolated mitochondria [165]; this allows the detection of poorly abundant metabolites and possibly mitochondrial dysfunction.

### 6.2. Mass Spectrometry

An alternative but complementary technique to NMR is mass spectrometry. It presents and combines several advantages. These techniques are more sensitive than classical NMR and cover a wider metabolite range. Mass spectrometry can be used alone with direct injection of the sample into the apparatus. Direct injection offers a rapid technique allowing the processing of a large number of samples (e.g. compatible with 96-well plates) [166]. However, sample complexity could be a problem and co-suppression or interference between peaks occurs regularly. This could be due to the matrix of the vehicle used (if any) [167,168]. Moreover, some technical problems could be encountered, such as contamination from different vehicles and sample diffusion [166]. However, optimized protocols are now available to circumvent such undesirable effects [166].

It is also possible to first separate the different metabolites before mass spectrometry through LC-MS, GC-MS or capillary electrophoresis (CE-MS). Once the different metabolites are separated, they can be led to a mass spectrometer to be analysed. Each technique presents different advantages and drawbacks. One of the most frequently used approaches is based on GC-MS because this technique has been used and validated in numerous publications [1,169,170]. Second, it allows simultaneous screening for different types of metabolites (including sugars, amino acids and lipids). Volatile metabolites are also compatible with GC-MS after a derivation step [171]. Another advantage of GC-MS is the availability of several databases allowing recognition of the spectra obtained by this technique. One of the best databases available is the NIST (National Institute of Standards and Technology) 14 Mass Spectral Library [172]. It contains more than 385,872 retention index values for 82,337 compounds (as of June 2014) and is compatible with both polar and apolar columns. This database also contains spectra for MS-MS (234,284 spectra) and an electron ionization library (276,259 spectra). Other free public databases have been developed, such as MassBank [173]. To date, this database contains more than 3251 spectra for metabolites and more than 4504 spectra for other chemicals [173]. Other databases, sometimes more complete, exist but are not free of charge. Amongst these paid, commercial databases is the Wiley Spectral Database [172]. In the 10th edition of this database, spectra are available for more than 736,000 unique compounds. Interestingly, some authors try to develop learning machines for the identification of mass spectra. In this study, a two-step process is described [174]. First, the developed algorithm creates a fingerprint prediction model from a large set of mass spectrometry data (from a public database; in this case, MassBank). Second, the algorithm matches the predicted fingerprint to a large database of molecules to associate the spectra with particular metabolites [174].

LC-MS presents different advantages over GC-MS. First, this technique is more sensitive than GC-MS. Second, it allows the detection of a wider molecular mass range. Third, derivation is not needed because it is mainly recommended for non-volatile metabolites. The resolution of metabolite separation is also greater and can be improved using ultra-performance liquid chromatography. However, unlike GC-MS, some metabolites are isolated/selected according to the column used. Therefore, more than one run is usually required to complete a particular study. Another drawback is that fewer databases are available for LC-MS spectra analysis than for GC-MS. As with NMR, this technique is compatible with labelling metabolites of interest. A method using 13C labelling and LC-MS to analyse metabolites in isolated mitochondria from murine skeletal muscle and NT2196 cancer cells was recently published [175].

The third technique, CE-MS, is still a minor technique compared to GC-MS and LC-MS. However, it presents better resolving power, requires less sample volume (which could be of interest with limited human samples) and has a shorter analysis time. It also allows the detection of a large number of metabolites (1000 at one time) [176]. Therefore, this is a promising technique for future metabolomics.

## 7. Impact of Metabolomics in the Field of Obesity, Type II Diabetes Mellitus and Metabolic Syndrome

### 7.1. Adipose Tissues and Obesity

Adipose tissue, which undergoes the most hypertrophic/hyperplasic modification in obese individuals (humans with a body mass index (BMI) ≥30 [177]) can be divided into three categories. First, the different anatomical localizations of white adipose tissue (WAT) are essentially composed of white adipocytes (characterized by a unilocular lipid droplet and a small stretched nucleus at the periphery of the cells), preadipocytes, blood vessels, inactive immune cells and fibroblasts [178]. WAT is mainly involved in handling of triglycerides (TG). The adipocytes can store energy as TG and mobilize them as FFAs to meet energy demands through a process called lipolysis [179]. In addition to their role in lipid metabolism, white adipocytes also play a strong role as endocrine cells. More than 400 proteins are secreted from and synthesized by adipocytes [180]. These proteins are known as adipokines or adipocytokines [181]. They were essentially discovered by proteomics studies analysing adipocyte secretomes in various conditions, such as adipogenesis in 3T3-L1 (murine preadipocyte differentiation) [182] and humans [183], at the basal state [184] or in response to an insulin challenge [183]. While the major roles of main adipokines have been reviewed elsewhere [185,186] very few have been characterized in detail thus far. Future studies should be conducted to address the role of these adipokines in adipocytes or any other cells targeted by adipocytes, such as immune, neuronal or muscle cells.

The second type of adipocytes is brown adipocytes. Their progenitors differ according to their localization. Interscapular brown fat derives from myogenic factor 5’s (Myf5) negative progenitors as skeletal muscle cells [187]. However, systemic brown adipocytes and white adipocytes share a common progenitor cell type expressing Myf5. Depending on the exposure to cytokines, the progenitor will express different proteins and differentiate either into a white adipocyte with exposure to bone morphogenetic proteins (BMP) 2 and 4 [188] or a brown adipocyte with exposure to BMP7 and fibroblast growth factors (FGF) 16 and 19 [189]. The differentiation between brown [189] and white adipocytes [188] has been reviewed in detail elsewhere. Brown adipocytes fulfil the distinct role of adaptive non-shivering-thermogenesis. This is allowed by the expression of UCP-1, which induces a mild mitochondrial uncoupling and heat production. It must be noted that brown adipose tissue has long been considered to be almost non-existent in adult humans [190]. However, recent studies have demonstrated both the presence and functionality of such adipose tissue in humans in response to cold exposure [184,191,192].

The third type of adipocytes is brite adipocytes. These cells possess the morphological and functional characteristics of brown adipocytes but are related to white adipocytes. As brown adipocytes, beige adipocytes express UCP-1 and can perform thermogenesis. The origin and importance of these cells is still highly debated and has been discussed comprehensively elsewhere [193].

As previously mentioned, white adipocytes can store energy as triglycerides. However, this capacity is limited. If exceeded, the excess lipids will be stored in other compartments such as the muscles, the liver and other non-adipose tissues [194]. This could lead to several deleterious consequences such as metabolic syndrome, which is a cluster of symptoms characterized by insulin resistance, T2DM (type 2 diabetes mellitus), dyslipidemia, chronic low-grade inflammation, atherosclerosis and hypertension [195]. However, obesity can also lead to the development of other diseases, including liver cirrhosis [196], heart failure [197] and some types of cancers [196,198].

### 7.2. Mitochondrial Dysfunction(s) in Obesity and Related Diseases

Mitochondria are thought to play a key role in the aetiology of obesity and T2DM. Fat accumulation and/or insulin resistance seem to affect mitochondria in several ways, including ROS production, mitochondrial structure, biogenesis and ATP production. We will discuss the role of these mitochondrial dysfunctions in the aetiology of obesity and related diseases.

First, numerous evidence shows increased mitochondrial ROS production [199] in response to obesity in animal models and humans. ROS production has been found to increase in the WAT of KKAy mice (a model of non-diabetic obesity), diet-induced obesity (DIO) mice and obese and type 2 diabetic *db*/*db*^−/−^ (knock-out for the leptin receptor) mice [200]. This can be explained by the increased expression and activity of NADPH oxidase and the decrease in the expression of antioxidant enzymes such as SOD1, SOD2, catalase and glutathione peroxidase (GPx) [200]. ROS production can also be explained by the presence of an elevated concentration of FFAs in the tissues and blood of obese individuals. FFAs can increase ROS production through the activation of NADPH oxidase [201]. ROS can also modulate the expression of genes involved in obesity in T2DM. The incubation of 3T3-L1 murine adipocytes in the presence of H_2_O_2_ mimics the situation observed during obesity in that it decreases adiponectin and PPARγ expression and increases the expression of pro-inflammatory molecules such as monocyte chemoattractant protein-1 (MCP-1), interleukine-6 (IL-6) and plasminogen activator inhibitor 1 (PAI-I) [200]. Incubation in the presence of antioxidant molecules, such as N-acetyl-cysteine, prevents these effects [200]. In humans, ROS production also seems higher in adipose tissue [200], lymphoblasts [202], monocytes [203] and the skeletal muscle [204] of obese patients. Interestingly, this increases more after stimulation with arachidonic acid [202], which suggests a direct role for this molecule and that food and its composition are directly responsible for the increased ROS production observed with obesity. In accordance with this hypothesis, a two-day fast decreased ROS production in human polymorphonuclear leucocytes [205]. Glucose, which is considerably increased in the blood of obese patients, also stimulates ROS production in rats’ liver cells or H9c2 rat myoblasts, which induces mitochondrial network fragmentation (glucose concentration: 50 mM) [206].

Other reports indicate that obesity can decrease mitochondrial biogenesis and the number of mitochondria. One piece of evidence for this is that *db*/*db*^−/−^ mice present a decreased mitochondrial population compared to lean non-diabetic wild-type C56/BL6 mice [207]. In general, mitochondrial abundance in fat deposits is inversely correlated with the weight of the mice. This could be explained by the decreased PGC-1β, PGC-1α, estrogen-related ERRα (ERReceptor α (ERRα) and PPARα activity observed in *db*/*db*^−/−^ mice [208]. In addition, a transcriptomic study performed on adipose tissue from *ob/ob*^−/−^ mice showed that the expression decreased in over 50% of the gene coding for mitochondrial proteins [209]. However, it seems that no T2DM mice model presents mitochondrial population alterations. The mitochondrial population of adipocytes in *ob/ob*^−/−^ mice is unaffected in relation to the number of mitochondria [207]. In humans, mitochondrial biogenesis alterations have also been found. Obese patients seem to present less subsarcolemmal mitochondria compared to lean subjects [207]. Interestingly, the mitochondrial DNA copy number is also inversely correlated with visceral fat accumulation in healthy young adults [210], which suggests that mitochondrial biogenesis could be affected in cell types other than adipocytes and skeletal muscle cells. This effect could be explained by the decrease in mitochondrial transcription factor A (mTFA/TFAM),) Nrf1 mRNA and protein levels induced by fat accumulation as measured in the cardiomyocytes of human patients [211]. This could also be due to elevated levels of tumour necrosis factor α (TNFα), which decreases the activity of endothelial nitric oxide synthase (eNOS) [212]. Decreased levels of transcription factors involved in mitochondrial biogenesis, such as PGC-1α and PGC-1β, are also found in the skeletal muscle of T2DM patients [213]. In another study performed on humans suffering from T2DM, forkhead box C2 (FOXC2), mTFA and TFAM subunit expression in respiratory complexes I, II, III and IV decreased in subcutaneous abdominal fat, thus suggesting a decrease in mitochondrial biogenesis [214]. Analysis of human diabetic kidney patients also revealed the down-regulation of PGC1α, which is a master regulator of mitochondrial biogenesis [215]. These results suggest a global decrease in the abundance and activity of mitochondria, which might be either the consequence or cause of kidney diseases [216]. Interestingly, T2DM patients are treated with rosiglitazone, which improves insulin sensitivity [217] and restores the expression of these proteins [214]. However, conflicting studies report no modification in terms of mitochondrial abundance in overweight and obese patients [218]. If obesity seems to have a strong effect on mitochondrial numbers, diabetes does not necessarily affect mitochondrial biogenesis *per se*; the effect is essentially dependent on the type of diabetes and the tissue considered. In hearts from the OVE26 mice model with type 1 diabetes, mitochondrial biogenesis is increased; increases are seen in the mitochondrial DNA copy number and in TFAM, which could be explained by the severe mitochondrial damages (reduced respiratory control ratio [ratio of mitochondrial respiration state 3 to state 4] due to a reduced state 3 rate and diminished GSH abundance) observed in this model [219].

In addition, alterations of mitochondrial morphology and the mitochondrial network can be observed in the mitochondria from obese individuals’ hepatocytes [220,221]. Alteration of the mitochondrial network could explain the reduced level of mitofusin 2 (Mfn2), which is a protein involved in the fusion of the OMM [222]) in Zucker rats [223] and the increased expression of dynamin-related protein 1 (Drp1) and mitochondrial fission protein 1 (Fis1), which are involved in OMM and IMM fusion, respectively, observed in HFD mice [224]. These modifications ultimately induce mitochondrial network fragmentation. The functional effect on mitochondrial dynamics has been confirmed in mice’s skeletal muscle cells that are transfected by electroporation in the presence of mitochondrial matrix-targeted photoactivable green fluorescent protein (mtPAGFP). The propagation of activated mtPAGFP after photo-activation is decreased in HFD mice compared to lean controls [224]. In accordance with studies performed on animal models, the Mfn2 level is also reduced in T2DM patients’ skeletal muscle [213,223]. Because mitochondrial (and mitochondrial network) morphology is linked with mitochondrial ATP production [223,225]; it is tempting to assume that ATP production is altered in obesity. In accordance with this hypothesis, ATP synthesis and mitochondrial respiration significantly decrease in the skeletal muscle cells of obese mice [224], obese humans and diabetic patients [226]. This is essentially because of a decrease in mitochondrial Complex I activity, as observed in overweight patients [211]. This suggests a functional significance of the mitochondrial alterations observed in response to obesity and T2DM.

In conclusion, mitochondrial dysfunction seems to have an important role in relation to the consequences of obesity, such as insulin resistance. However, recent reports have suggested that mitochondrial dysfunction is not necessarily required to induce insulin resistance (and possibly other obesity-related diseases). Some conditions that induce insulin resistance do not always affect mitochondrial function. Conversely, alterations of mitochondrial function do not always induce insulin resistance. Mitochondrial respiration inhibition by rotenone is sufficient to induce insulin resistance (as measured by decreased insulin-stimulated glucose uptake) in 3T3-L1 murine adipocytes but does not affect FAO hepatocytes’ insulin sensitivity (even if the basal and insulin-stimulated glucose uptakes decrease) [227]. In addition, anti-diabetic drugs to improve insulin sensitivity, such as rosiglitazone, induce mitochondrial dysfunction. Following treatment of 3T3-L1 adipocytes with rosiglitazone, mitochondrial respiration decreased and glycolysis increased [227]. ROS production was not affected by the treatment. Interestingly, basal glucose uptake significantly increased but insulin-stimulated glucose uptake was not affected, which suggests that these drugs’ anti-diabetic effect could be cell-specific [227]. Similar results have been obtained on intact hepatocytes and isolated mitochondria from rats’ skeletal muscle (inhibition of states I and III of mitochondrial respiration) [228]. However, a decrease in ROS mitochondrial production was observed under these circumstances, which emphasises the cell-specific effect of this class of drug [228,229]. In contrast, in humans treated with rosiglitazone, increased expression of mitochondrial ETC components was observed in patients with high oxygen consumption (VO_2_ over 28.1 mL/kg/min) [230]. However, the anti-diabetic function of rosiglitazone was similar in both types of patients, thus suggesting that the gain in mitochondrial function is not required for insulin resistance improvement. The glitazones’ effects on ETC components are likely due to the direct binding of glitazones on mitochondrial complex I. It has been recently suggested that, glitazones can disassemble disassembly complex I following binding, thereby reducing complex I activity and ATP production, at least in human HepG2 hepatocytes [229]. Similar results for mitochondrial complex I activity have been obtained with other anti-diabetic drugs (phenformin and biguanides) belonging to another class of anti-diabetic drugs [227].

In conclusion, despite numerous evidence of implication of mitochondrial dysfunction(s) in the onset of obesity and its consequences, the role of these different types of mitochondrial dysfunction could be different according to the cell type considered and does not seem to be necessarily required to observe insulin resistance. Therefore, further studies are still required in order to ascertain the role of mitochondrial dysfunction in these diseases.

We also emphasize that mitochondrial dysfunction is usually evaluated by measuring ATP content or production, mitochondrial potential membrane or ROS production. However, mitochondrial dysfunction also includes other dysfunctions, such as perturbations in mitochondrial metabolism and metabolite concentration. Therefore, metabolomic studies offer a unique opportunity to screen for metabolites and evaluate the pertinence of such mitochondrial dysfunctions in obesity. As more people are affected by obesity and its associated disorders, it is necessary to detect the possible development of these consequences to treat them as quickly as possible. The method of choice should be non-invasive, fast, affordable and reliable. Once again, metabolomics meet all these requirements, as do other “-omics” techniques such as the lipidomic, transcriptomic and proteomic approaches. Next, we will summarize what metabolomics, which is the most integrative “-omics” approach, has brought to the obesity research field. The principal modifications are presented in Table 2.

**Table 2:** Summary of metabolites found as modified in response to insulin resistance.

Class of metabolites	Metabolite(s) identified	Trend in insulin resistance	Species	Tissue or cell-type	Reference
Free fatty acids	Myristate, palmitate, stearate, oleate, linoleate, arachidonate	Increase	Human	Blood	[231]
	3-carboxy-4-methyl-5-propyl-2-furanpropanoic acid	Increase	Human	Serum	[232]
Carnitines	Dodecenoyl carnitine, tiglyl carnitine, tetradecenoyl carnitine, lauroyl carnitine, propionyl carnitine	Increase	Mice	Blood	[233]
	Acetylcarnitine, proprionylcarnitine, deoxycarnitine	Increase	Mice	Blood	[234]
	Proprionyl carnitine, isovaleryl/2-methylbutyryl carnitine, hexanoyl carnitine, octenoyl carnitine	Increase	Human	Blood	[231]
	Carnitine	Increase	Mouse	Serum	[235]
LysoLyso-glycerophospholipids	Lysophosphatidylethanolamine (20:1, 20:2, 22:4) Lysophosphocholine (20:5, 18:2, 18:1) Lysophosphatidyl inositol (20:4) Lysophisphatidylserine (20:0) lysophosphatidic acid (18:2)	Increase	Human	Blood	[236]
	Lysophosphocholine (16:1)	Decrease	Mice	Blood	[237]
	Lysophosphocholine (22:4)	Increase	Mice	Blood	[237]
	Lysophosphocholine	Increase	Human	Serum	[238]
	Diacyl-phosphatidylcholines Lysophosphocholines	Decrease	Mouse	Serum	[239]
	Lysophosphatidylcholine Phosphatidylserine	No change	Mouse	Liver	[237]
	Lysophosphatidylcholine (18:0)	Increase	Mouse	Serum	[240]
Amino acids	Serine, glycine, arginine	Decrease	Mouse	Serum	[239]
	Alanine, arginine, glycine, isoleucine, methionine, ornithine, serine	Decrease	Mouse	Serum	[235]
	Valine, leucine, isoleucine, phenylalanine, tyrosine, glutamate/glutamine, aspartate/asparagine, arginine	Increase	Human	Serum	[231]
	Citrulline, histidine, methionine, ornithine, proline, serine, tryptophan	No change	Human	Serum	[231]
TCA cycle	Succinate	Increase	Mouse	Serum	[235]
	Citrate, glucoxe, glycolate, lactate, pyruvate	Decrease	Mouse	Serum	[235]
Ketone bodies	Acetoacetate, acetoneµ	Decrease	Mouse	Serum	[235]
	2-hydroxybutyric acid	Increase	Human	Serum	[169,231]
Others	Hydroxysphingomyelin	Decrease	Mouse	Serum	[239]

### 7.3. Metabolomic and Lipidomic Studies

One of the most important classes of metabolites in the field of obesity-related pathologies and mitochondrial dysfunction is lipids. First, obese patients present ectopic lipid storage in several tissues, including adipocytes, macrophages, skeletal muscle cells and hepatocytes [241]. Thus, lipid concentrations are highly modified in these conditions. Second, lipids are essentially catabolized by the mitochondria and the peroxisomes for very long fatty acids. Therefore, an excessive accumulation of lipid-derived molecules could be a possible indicator of mitochondrial dysfunctions that could be involved in the aetiology of obesity-linked diseases.

It seems particularly important to study two classes of lipids: FFAs and acylcarnitines. An interesting metabolomic study performed on the muscles, serums and WAT of rats exposed to a high-fat diet, a “cafetariat” diet and a control diet suggests that saturated fatty acids (stearate, palmitate and myristate) are elevated in virtually every tissue in obese rats [233]. An increased blood concentration of FFAs (such as myristate, palmitate, stearate, oleate, linoleate and arachidonate) was also observed in obese humans [231]. Several fatty acids (including total saturated, monounsaturated and polyunsaturated) were also elevated in the livers of HFD C57BL/6 mice [237], which explains the hepatic steatosis observed in these conditions. However, α-linolenic acid decreased under the same conditions. This evidence shows that obesity induces strong modifications in fatty acid metabolism. This global increase in FFA levels could have several deleterious effects, especially on mitochondria. These deleterious effects include mitochondrial uncoupling [242], decreased mitochondrial ATP production leading to increased glycolysis rates and increased ROS production [115]. All these effects have been recapitulated through the direct incubation of H9c2 cardiac myocytes in rats and C2C12 murine skeletal muscle cells in the presence of fatty acids such as palmitate [242,243]. Monitoring different FFAs levels is thus a good method to identify the possible consequences of mitochondrial dysfunctions. However, increased FFA is a common event in obesity, even in non-insulin-resistant obese individuals where no mitochondrial dysfunction is expected. Therefore, other lipids must be considered to discover the potential biomarkers of mitochondrial dysfunction. One interesting class of candidates is acylcarnitines, which are FFAs coupled to carnitine. Levels of acylcarnitines (such as dodecenoyl carnitine, tiglyl carnitine, tetradecenoyl carnitine, lauroyl carnitine and propionyl carnitine) are higher in HFD rats [233]. Increased levels of other acylcarnitines (acetylcarnitine, proprionylcarnitine and deoxycarnitine) have also been found in HFD mice [234]. The increase in some circulating acylcarnitines (proprionyl carnitine, isovaleryl/2-methylbutyryl carnitine, hexanoyl carnitine and octenoyl carnitine) has also been confirmed in obese patients [231]. The high concentration of acylcarnitines in mitochondria is often associated with mitochondrial dysfunction [233] and could represent insufficient β-oxidation [244]. Interestingly, these elevated concentrations are correlated with higher body mass in rats, glucose concentrations, an adipose tissue inflammatory state (presence of crown-like structure in adipose tissue, recruitment of pro-inflammatory M1 macrophages), conditions associated with mitochondrial dysfunction and metabolic syndrome [233]. The authors have also identified several extra molecules that are present in adipose tissue lesions in high amounts: laurate and lauroyl carnitine [233]. The authors also successfully demonstrated the physiological role of these molecules by demonstrating pro-inflammatory activation in bone-marrow macrophages, which is a process mediated by AMPK inactivation and increased pro-inflammatory cytokines such as IL-3, IL-12, IL-16, IL-23, CXC chemokine ligand 11 (CXCL11), CXCL13, CC-chemokine ligand 5 (CCL5) and CCL17 [233]. The accumulation of acylcarnitines is clearly associated with insulin resistance. Clearing these lipids (through physical exercise or the knock-out of malonyl-CoA decarboxylase) improves insulin resistance in mice [244].

Lyso-glycerophospholipids is another interesting class of lipids. These molecules are membrane-derived phospholipids formed in response to a cell stimulus [245]. Lyso-glycerophospholipids are often considered to be signalling and pro-inflammatory molecules [246]. The synthesis and roles of these molecules in chronic inflammatory diseases have been reviewed in detail elsewhere [245]. Lyso-glycerophospholipids have also been linked to mitochondrial dysfunctions. First, the insertion of lysophospholipids (one class of lyso-glycerophospholipids) in the mitochondrial membrane promotes its permeabilization [247] and inhibits the activity of mitochondrial COX1 and glycerolphosphate dehydrogenase [248]. Concentrations of 0.75 mg of lysophospholipids/mg protein can completely alter the mitochondrial membrane integrity and induce the complete release of malate dehydrogenase from the mitochondrial matrix [248]. Lysophospholipids are also often considered to be cell death effectors and to play an important role in lipotoxicity [249]. This class of lipids thus seems to be particularly interesting in relation to obesity and mitochondrial dysfunctions.

Several pieces of evidence indicate the possible alteration of lyso-glycerophospholipid metabolism in obesity and its related diseases. By comparing the metabolomes (using LC-QTOF/MS, GC-Q/MS and CE-TOF/MS) of 20 women suffering from gestational diabetes with the metabolomes of 20 healthy women, the authors found increases in some lyso-glycerophospholipids such as lysophosphoethanolamine (LPE) (20:1), LPE (20:2), LPE (22:4), lysophosphatidylcholine (LPC) (20:5), LPC (18:2), LPC (18:1), lysophosphatidylinositol (LPI) (20:4), LPS (20:0), (LPA) (18:2) [236]. Based on the correlation between lyso-glycerophospholipids and obesity/weight, a study using flow injection analysis/ thermospray mass spectrometry (FIA-MS) to analyse serums from obese children who lost weight (compared with children with stable weights) showed increased levels of lysophosphocholines [238]. However, some lysophosphatidylcholines could be differentially affected by obesity as seen in HFD C57BL/6 mice. Lysophosphatidylcholine (16:1) seemed to decrease when the level of lysophosphatidylcholine (22:4) increased [250]. The elevated lysophosphocholine concentration in insulin-resistant patients was reported to be associated with the number of servings of full-fat dairy foods [251]. However, every class of lysoglycerophospholipids does not seem to be affected in the same way by obesity and/or diabetes; these effects could depend on the model considered. In a study using LC/MS/MS, the level of twenty two diacyl-phosphatidylcholines, two lysophosphocholines and three carnitines was lower in serum from the Berlin Fat Diabetic Mouse Inbred line, whereas higher concentrations of other metabolites, such as amino acids (serine, glycine and arginine) and hydroxysphingomyelin, were found [239]. In accordance with this finding, lysophosphatidylcholine and phosphatidylserine level was not affected in the livers of HFD C57BL/6J mice [237]. However, the association between lower phosphatidylcholines and obesity was confirmed in mice by another study conducted with FVB/N mice [240]. In conclusion, it seems that obesity is clearly associated with modifications to lyso-glycerophospholipids levels (which could represent a risk of mitochondrial dysfunction) even though some discrepancies were observed in some models. More studies are thus required to assess the role of this class of lipids in obesity and its related diseases, especially their role in mitochondrial dysfunctions.

Other intermediates and metabolites of lipid metabolism are affected by obesity and seem to be linked to mitochondrial dysfunctions. Serums from patients with either gestational diabetes or T2DM showed major alterations in fatty acid metabolites compared to the controls, especially in furan fatty acid metabolites such as 3-carboxy-4-methyl-5-propyl-2-furanpropanoic acid (CMPF) [232]. An elevation of CMPF in mice results in glucose intolerance, impaired insulin secretion and decreased glucose uptake by causing mitochondrial dysfunction (decreases in the mitochondrial potential membrane and increases in mitochondrial ROS production) in β-cells that then impair the activity of transcription factors controlling insulin biosynthesis [232]. A beneficial effect from CMPF-induced pancreatic β-cell dysfunction could be obtained by blocking the CMPF transporter (OAT3) or an antioxidant treatment [232].

Because diabetes is strongly associated with perturbations in lipid metabolism, one can postulate that improved insulin sensitivity could improve this metabolism. However, although they can clearly alleviate T2DM, glitazones are associated with an increased risk of hepatic steatosis. This could be due to their side effects on lipid metabolism, as demonstrated in a lipidomic study performed on obese mice [252]. First, glitazones strongly affect the transcript level of several enzymes that handle lipid metabolism, including increases in fatty acid synthase, lipoprotein lipase, fatty acid translocase and acetyl-CoA oxidase, thus suggesting a global increase in lipid metabolism under these circumstances [252]. In accordance with these results, levels of several lipids are affected in a tissue-specific manner. Triglyceride levels decrease in the liver while they increase in plasma and remain unaffected in the heart or adipose tissue. Phosphatidylcholines globally decrease in plasma membranes but increase in hepatic and cardiac tissues [252], while lysophosphatidylcholines are unaffected no matter what tissue is studied. Cardiolipins remain globally unaffected in plasma but decrease in the heart [252]. Finally, phosphatidylserines and phosphatidylethanolamines globally increase in the liver and heart, while their levels decrease in blood [252]. Another study reported corrected levels of long-chain fatty acids in serum from rosiglitazone-treated patients [253]. All these studies clearly showed that even if levels of some glycerophospholipids (phosphatidylcholines, phosphatidylserines, and phosphatidylethanolamines) and long-chain fatty acids are corrected by glitazones, others (such as lysophosphatidylcholines or triglycerides) are not affected. Therefore, since insulin resistance is clearly alleviated by this treatment, one can postulate that some lipids that are modified by diabetes do not clearly play a role in this disease. Second, it also shows that glitazones have critical side effects. Therefore, these molecules must be used with caution; when possible, their use must be associated with metabolite levels monitoring in patients treated with glitazones. Biguanides also have a clear effect on lipid profiling. Metformin treatment in diet-induced-obese mice increases the urinary levels of some phosphatidylcholines (15:0, 16:0, 18:0) [254], but these results were challenged in another study [255]. In addition to this role, metformin also seems able to limit palmitate stress induced in INS-1 beta cells by decreasing endoplasmic reticulum stress (through decreases in C/EBP homologous protein (CHOP) activation, the phosphorylation of eIF2α and Janus kinases (JNK)) [256]. Amino acid metabolism also seems to be affected by obesity. Some authors found significant modifications in some amino acid levels (leucine, alanine, arginine, lysine and methionine) in C57/BL6 mice with leptin gene deficiencies (*ob*/*ob* mice) compared to wild-type mice [235]. These authors also found modifications in TCA cycle metabolites (pyruvate, citrate and glycolate), ketone bodies (acetoacetate and acetone), creatine metabolism and lipid metabolism (cholesterol and carnitine). BCAAs also represent a target of choice in evaluating the possible induction of mitochondrial dysfunctions in obesity and its related diseases. BCAA catabolism is performed in the mitochondrial matrix. Therefore, modifications of these metabolites’ levels could be an indicator of mitochondrial functionality. A retrospective study performed on 50 patients found significantly higher BCAA blood concentrations in patients suffering from primary mitochondrial disease or from pyruvate dehydrogenase deficiency [257]. BCAA catabolism products can also affect mitochondrial functionality, especially FFA β-oxidation rates [258]. This evidence suggests a possible link between increased BCAA concentrations and mitochondrial functionality, at least under certain circumstances.

In accordance with this role and the possible implication of mitochondrial dysfunction in obesity, obese humans also present increased circulating levels of BCAAs (valine, leucine and isoleucine) and other amino acids (phenylalanine, tyrosine, glutamate/glutamine, aspartate/asparagine and arginine) [231]. However, concentrations of other amino acids remain unaffected (citrulline, histidine, methionine, ornithine, proline, serine and tryptophan) [231]. This suggests that only some are important in obesity. Plasma concentrations in BCAAs also decrease during weight loss and are correlated with insulin sensitivity in humans, thus indicating that BCAAs are good biomarkers in evaluating the evolution of insulin sensitivity in humans [259]. This accumulation of BCAAs could be due to the decreased expression of enzymes involved in their catabolism, which was demonstrated in a study performed on mitochondria isolated from the skeletal muscle of obese and insulin-resistant patients [204]. However, conflicting results showed decreased levels of BCAAs in WAT from HFD mice, which has been associated with increased BCAA catabolism [234]. Therefore, the precise role of BCAAs in obesity and insulin resistance is still controversial. Indeed, if BCAAs are linked to the development of insulin resistance, we would expect to see increased insulin resistance in rats exposed to BCAAs. To confirm this hypothesis, a study was performed on rats exposed to an HFD and complemented with BCAAs. Interestingly, these rats were equally insulin resistant to HFD rats [231]. BCAAs do not affect rats fed with a classic diet [231]. In addition, BCAA complementation does not have any inhibitory effect on the increased levels of acylcarnitines observed in HFD rats’ skeletal muscle, which is a previously seen indicator of mitochondrial dysfunction [231]. However, insulin resistance observed in BCAA-HFD rats seems different compared to HFD rats. This treatment seems to induce mTOR (Ser2448) and IRS1 (Ser307) phosphorylation and increase JNK protein levels [125]. Moreover, treatment with rapamycin, an mTOR inhibitor, seems to alleviate insulin resistance only in BCAA-HFD-fed rats [231]. Supporting the role of BCAAs in obesity, the knock-out of the BCATm gene seems to increase BCAA levels (as seen in obesity); however, mice are surprisingly protected against HFD-induced obesity and have better glucose and insulin tolerance [260]. Another study reported that middle-aged mice fed with BCAAs had prolonged lifespan, decreased ROS production (due to the increased expression of genes encoding antioxidant enzymes), increased mitochondrial biogenesis and increased sirtuin-1 expression in cardiac and skeletal muscle [41]. In addition, treatment with rosiglitazone also increased BCAA levels in the plasma of treated patients [253]. Similar results were observed in humans’ BCAA blood levels in response to metformin treatment [261]. However, conflicting results were obtained in another study performed on adipose tissue from Zucker rats; an increased expression of enzymes involved in BCAA catabolism was found [262]. The link between BCAAs, obesity and insulin resistance is unclear and must be investigated further. This demonstrates the possibility that the biology of BCAAs in obesity could also differ between mice, rats and humans. This aspect merits more attention in the future. Despite its unclear role in insulin sensitivity, BCAAs also seem to play a role in the development of coronary disease, which is another possible consequence of obesity. A link has been demonstrated between increased serum BCAA concentrations in humans and waist circumference, blood pressure, fasting blood glucose, TG, apolipoprotein B (apoB), apoB/apoAI ratio, apoCII and apoCI levels, which are important risk factors in coronary diseases [263].

Another class of metabolites of special interest is ketone bodies. These molecules are produced in the mitochondrial matrix during times of energy demand and glucose deprivation. Alterations of this metabolism have been associated with obesity [169]. A study performed on 163 Chinese patients (ranging from 25–70 years old) found three metabolites associated with the possible development of metabolic syndrome: 2-hydroxybutyric acid (a ketone body), inositol and glucose [169]. Another study found similar results regarding the ketone body levels. In a study performed on 74 obese humans, β-hydroxybutyrate levels were elevated in obese individuals [231]. The accumulation of 3-hydroxybutyrate could be a direct effect of FFA exposure. The incubation of isolated mitochondria from the skeletal muscle of C57Bl/6J mice in the presence of palmitate induces an accumulation of 3-hydroxybutyrate in the mitochondrial matrix [264]. However, the possibility of using 3-hydroxybutyrate as a biomarker for insulin resistance is compromised by results showing that metformin treatment of patients also increases serum concentrations of this metabolite and acetoacetate [255]. Therefore, increased levels of 3-hydroxybutyrate might be caused by the mitochondrial dysfunction induced by this drug and may not be related to the onset of insulin resistance.

Chronically high glucose blood concentrations are also a hallmark of insulin resistance [265]. To elucidate the consequences of this chronic exposure to glucose, a metabolomic study was performed on INS-1cells exposed to high and low glucose concentrations using GC/MS. The authors found increases in some TCA cycle components (citrate, succinate, α-ketoglutarate, fumarate and isocitrate) in response to high glucose conditions. These modifications were accompanied by increased glycolysis rates, mitochondrial respiration and mitochondrial proton leaks and indicate a possible mitochondrial dysfunction [266]. However, we can still question the importance of these modifications in the aetiology of insulin resistance. Treatments alleviating insulin resistance, such as glitazones, also up-regulate glycolysis and lactate and acetate production, which are associated with increased anaerobic glycolysis and fatty acid β-oxidation. As demonstrated in humans, biguanides also considerably decrease the abundance of virtually every TCA cycle intermediate [267]. This is in accordance with the mitochondrial dysfunction induced by these molecules.

In addition to lipids and sugars, organic anions/cations also seem to be good biomarkers of the potentially detrimental consequences of obesity. GC-MS quantification of 94 metabolites recovered from the urine of a cohort of T2DM patients (with or without kidney disease) compared to a healthy control group revealed that obesity affected 13 organic anions [216]. Furthermore, the analysis of gene expression from human diabetic nephropathy revealed a lower expression of the genes encoding organic anion transporters (OAT-1 and OAT-3), which could explain the differences observed. In accordance with this hypothesis, twelve OAT-transported organic anions were related to mitochondrial metabolism. In addition to organic anions, organic cations could also play in important role in diabetes. Elevated protein levels of organic cation transporter-1 (OCT-1) were found in the livers of HFD mice [268]. Interestingly, metformin can inhibit OCT-1 [269]. Moreover, loss of OCT-1 reduces TG liver content in both lean and *ob*/*ob* obese mice, which is caused by the inhibition of thiamine uptake, AMPK phosphorylation and acetyl-CoA carboxylase activity [269]. Therefore, organic cations should be considered as potential biomarkers for insulin resistance.

Despite the great advances provided by metabolomic studies in the obesity field, we still must note that most of these studies were performed on plasma, blood, whole cells or tissue lysates. However, the nature of such samples is complex and could reduce the number of metabolites that can be detected. Mitochondrial metabolites (and thus the molecular signatures of mitochondrial dysfunction) can remain undetected when these analyses are performed on cells/tissues. Therefore, some authors propose directly analysing the metabolites of isolated mitochondria. An excessive palmitate concentration is often associated with very high rates of FFAs β-oxidation and mitochondrial overload with incomplete lipid oxidized products; this condition is known to induce mitochondrial dysfunction and lead to insulin resistance [244]. Using GC-MS, Seifert *et al.* studied the effect of palmitate on skeletal muscle cells and its consequences on the composition of a metabolite mixture in a buffer and a matrix of mitochondrial fractions [264]. They found that palmitate leads to changes in TCA components, such as increased citrate and α-ketoglutarate and decreased succinate and malate; this suggests the possible induction of a mitochondrial dysfunction [264]. Interestingly, another study performed on mice and humans isolated mitochondria that were incubated in the presence of palmitoyl carnitine, palmitoyl-CoA and oleoyl-CoA and reported the stimulation of mitochondrial ATP production [270]. However, if their concentrations exceed 5 µM, mitochondrial dysfunction occurs. ATP synthesis is inhibited and a depolarization of the mitochondrial membrane is observed, thus suggesting these molecules finely tuned regulation of mitochondrial ATP production [270]. This observation also suggests that mitochondrial dysfunction(s) will occur only if FFA concentrations are excessive. Alterations in the TCA cycle during mitochondrial dysfunction were shown by another study performed on isolated mitochondria incubated in the presence of ^13^C-radiolabeled substrates and rotenone, which is a mitochondrial complex I inhibitor [70]. Metabolite analysis was performed with GC/MS. A clear mitochondrial dysfunction was observed in this study along with increased citrate and decreased oxaloacetate, succinate, α-ketoglutarate and aspartate in the mitochondrial matrix [175]. However, this study was performed in breast cancer cells (NT2196); therefore, the modifications found might not reflect the biology of normal cells. However, these findings support the fact that TCA cycle component modification is a major cause of mitochondrial dysfunction(s).

We also must remember that a metabolome can be strongly influenced by several factors, such as gender, food intake, physical exercise, ethnicity and age. A recent study performed on 192 overweight adolescents (ranging from 12–15 years old) demonstrated many differences between boys and girls [271]. For instance, girls present higher urinary excretions of citrate, creatinine, hippurate and phenylacetylglutamine and higher plasma levels of phosphatidylcholine and unsaturated lipids compared to boys of the same age [271]. The pubertal development stage also affects the metabolome. It was positively related to urinary creatinine excretion and negatively related to urinary citrate levels [271]. However, the authors of this study could not show that physical exercise affected the metabolome [271]. Intracytoplasmic sperm injection (ICSI) could also affect the metabolome. In a study performed on 42 children born after ICSI and 42 naturally conceived children, urea and thyroid hormone T3 levels were lower in ICSI children [272]. More than 40 metabolites were also significantly elevated in ICSI children, including C-reactive protein (CRP) and glucose; TCA cycle components, such as citrate and isocitrate; and lipids, such as myristate and cholesterol [272]. In conclusion, we must be very careful when suggesting a metabolite as a potential biomarker of obesity and/or mitochondrial dysfunction. Ideally, the influence from any factors, such as gender, age, ethnicity and method of conception, must be corrected before making any conclusion relative to the significance of the biomarker considered.

We have seen that metabolomics represent an attractive experimental approach that can be used for prognostics and diagnostics. However, they require specific instruments, time and qualified people to ensure the quality and reproducibility of the results. Therefore, some authors proposed another strategy: implanting an external device into the patient’s body [273]. This would allow the real-time analysis of the metabolite set of interest. However, the presence of a foreign material could induce a pro-inflammatory process and therefore alter the concentrations of some metabolites of interest. By using microdialysis to model implanted sensors, the study demonstrated that glucose, lactate and urea concentrations rise over time following implantation [273]. This has been associated with the pro-inflammatory response induced by the implanted capillary. However, increased levels were also found for other metabolites, such as glycerol and pyruvate, retrieved at high concentrations in T2DM patients [273]. This confirms the possibility of real-time screening of some metabolites of interest in patients, thus allowing rapid treatment when necessary.

## 8. Conclusions

We have seen that mitochondria are important players in many cellular metabolic pathways, including the TCA cycle, FFA β-oxidation, glutaminolysis, BCAA catabolism and steroidogenesis. Because of their central role in metabolism, dysfunctions in this organelle have many deleterious effects. Mitochondrial dysfunction(s) can lead to the development of several pathologies, including obesity-related diseases such as atherosclerosis and metabolic syndrome. However, the role of mitochondria in these pathologies is still highly debated and must therefore be studied in more detail in the future.

We have also seen that mitochondrial dysfunctions are often associated with specific metabolite signatures. Therefore, it is possible to use approaches such as lipidomics and metabolomics to detect the early signature of mitochondrial dysfunctions. If these signatures could be validated for particular diseases, it would therefore be possible to detect these markers early in patients before the onset of symptoms. This would allow a faster and more appropriate treatment of mitochondria-associated diseases. Therefore, future studies must be dedicated to the validation of such biomarkers (ideally both in animal models and humans). Metabolomic approaches seem to be ideal for achieving this goal. However, because every metabolomic approach uses different techniques and instruments, the results are often limited to the detection of only a few classes of metabolites. Different and complementary techniques must therefore be used to ensure the most complete and comprehensive metabolic profile and alterations.

One could also imagine the possibility of using mild mitochondrial uncoupling in adipocytes as a suitable treatment for obesity [274]. Indeed, the increase in energy expenditure would allow decreased TG content in obese individuals’ adipose tissues and would subsequently limit the consequences of this pathological state [275]. To achieve this goal, the use of some metabolites might be envisaged. Indeed, we have seen that almost any class of metabolites can interfere with the regulation and/or the activity of UCPs. It is therefore tempting to think of using these metabolites to regulate the activity of UCPs. However, because few mechanisms are available for this regulation, further studies are needed to identify metabolites that can regulate UCP expression and/or activity.

## References

[1] Beger R.D. (2013). A review of applications of metabolomics in cancer. Metabolites.

[2] Fiehn O. (2001). Combining genomics, metabolome analysis, and biochemical modelling to understand metabolic networks. Comp. Funct. Genom..

[3] Nicholson J.K., Lindon J.C., Holmes E. (1999). “Metabonomics”: Understanding the metabolic responses of living systems to pathophysiological stimuli via multivariate statistical analysis of biological nmr spectroscopic data. Xenobiotica.

[4] Robertson D.G. (2005). Metabonomics in toxicology: A review. Toxicol. Sci..

[5] Liesenfeld D.B., Habermann N., Owen R.W., Scalbert A., Ulrich C.M. (2013). Review of mass spectrometry-based metabolomics in cancer research. Canc. Epidemiol. Biomarkers Prev..

[6] Vermeersch K.A., Styczynski M.P. (2013). Applications of metabolomics in cancer research. J. Carcinog..

[7] Gray M.W., Burger G., Lang B.F. (2001). The origin and early evolution of mitochondria. Genome Biol..

[8] Acin-Perez R., Fernandez-Silva P., Peleato M.L., Perez-Martos A., Enriquez J.A. (2008). Respiratory active mitochondrial supercomplexes. Mol. Cell.

[9] Schagger H., Pfeiffer K. (2000). Supercomplexes in the respiratory chains of yeast and mammalian mitochondria. EMBO J..

[10] Schagger H., Pfeiffer K. (2001). The ratio of oxidative phosphorylation complexes i-v in bovine heart mitochondria and the composition of respiratory chain supercomplexes. J. Biol. Chem..

[11] Strogolova V., Furness A., Robb-McGrath M., Garlich J., Stuart R.A. (2012). Rcf1 and rcf2, members of the hypoxia-induced gene 1 protein family, are critical components of the mitochondrial cytochrome bc1-cytochrome c oxidase supercomplex. Mol. Cell. Biol..

[12] Ikeda K., Shiba S., Horie-Inoue K., Shimokata K., Inoue S. (2013). A stabilizing factor for mitochondrial respiratory supercomplex assembly regulates energy metabolism in muscle. Nat. Commun..

[13] Wenz T., Hielscher R., Hellwig P., Schagger H., Richers S., Hunte C. (2009). Role of phospholipids in respiratory cytochrome bc (1) complex catalysis and supercomplex formation. Biochimica Biophys. Acta.

[14] Bottinger L., Horvath S.E., Kleinschroth T., Hunte C., Daum G., Pfanner N., Becker T. (2012). Phosphatidylethanolamine and cardiolipin differentially affect the stability of mitochondrial respiratory chain supercomplexes. J. Mol. Biol..

[15] Bazan S., Mileykovskaya E., Mallampalli V.K., Heacock P., Sparagna G.C., Dowhan W. (2013). Cardiolipin-dependent reconstitution of respiratory supercomplexes from purified saccharomyces cerevisiae complexes iii and iv. J. Biol. Chem..

[16] McBride H.M., Neuspiel M., Wasiak S. (2006). Mitochondria: More than just a powerhouse. Curr. Biol..

[17] Tait S.W., Green D.R. (2010). Mitochondria and cell death: Outer membrane permeabilization and beyond. Nat. Rev. Mol. Cell Biol..

[18] Kubli D.A., Gustafsson A.B. (2012). Mitochondria and mitophagy: The yin and yang of cell death control. Circ. Res..

[19] Rizzuto R., de Stefani D., Raffaello A., Mammucari C. (2012). Mitochondria as sensors and regulators of calcium signalling. Nat. Rev.Mol. Cell Biol..

[20] Williams G.S., Boyman L., Chikando A.C., Khairallah R.J., Lederer W.J. (2013). Mitochondrial calcium uptake. Proc. Natl. Acad. Sci. USA.

[21] Tait S.W., Green D.R. (2012). Mitochondria and cell signalling. J. Cell Sci..

[22] Levine A.J., Puzio-Kuter A.M. (2010). The control of the metabolic switch in cancers by oncogenes and tumor suppressor genes. Science.

[23] Cardaci S., Ciriolo M.R. (2012). Tca cycle defects and cancer: When metabolism tunes redox state. Int. J. Cell Biol..

[24] Lazarow P.B. (1978). Rat liver peroxisomes catalyze the beta oxidation of fatty acids. J. Biol. Chem..

[25] Foster D.W. (2004). The role of the carnitine system in human metabolism. Ann. NY Acad. Sci..

[26] Bartlett K., Eaton S. (2004). Mitochondrial beta-oxidation. Eur. J. Biochem..

[27] Cho S.Y., Kim J.H., Paik Y.K. (1998). Cholesterol biosynthesis from lanosterol: Differential inhibition of sterol delta 8-isomerase and other lanosterol-converting enzymes by tamoxifen. Mol. Cells.

[28] Cotter D.G., Schugar R.C., Crawford P.A. (2013). Ketone body metabolism and cardiovascular disease. Am. J. Physiol. Heart Circ. Physiol..

[29] Meng M., Chen S., Lao T., Liang D., Sang N. (2010). Nitrogen anabolism underlies the importance of glutaminolysis in proliferating cells. Cell Cycle.

[30] Nicklin P., Bergman P., Zhang B., Triantafellow E., Wang H., Nyfeler B., Yang H., Hild M., Kung C., Wilson C. (2009). Bidirectional transport of amino acids regulates mtor and autophagy. Cell.

[31] Smith D.D., Campbell J.W. (1988). Distribution of glutamine synthetase and carbamoyl-phosphate synthetase i in vertebrate liver. Proc. Natl. Acad. Sci. USA.

[32] Boza J.J., Moennoz D., Bournot C.E., Blum S., Zbinden I., Finot P.A., Ballevre O. (2000). Role of glutamine on the de novo purine nucleotide synthesis in caco-2 cells. Eur. J. Nutr..

[33] Cory J.G., Cory A.H. (2006). Critical roles of glutamine as nitrogen donors in purine and pyrimidine nucleotide synthesis: Asparaginase treatment in childhood acute lymphoblastic leukemia. In Vivo.

[34] Mouilleron S., Badet-Denisot M.A., Golinelli-Pimpaneau B. (2006). Glutamine binding opens the ammonia channel and activates glucosamine-6p synthase. J. Biol. Chem..

[35] Dang C.V. (2010). Glutaminolysis: Supplying carbon or nitrogen or both for cancer cells?. Cell Cycle.

[36] Metallo C.M., Gameiro P.A., Bell E.L., Mattaini K.R., Yang J., Hiller K., Jewell C.M., Johnson Z.R., Irvine D.J., Guarente L. (2012). Reductive glutamine metabolism by idh1 mediates lipogenesis under hypoxia. Nature.

[37] Brosnan J.T., Brosnan M.E. (2006). Branched-chain amino acids: Enzyme and substrate regulation. J. Nutr..

[38] Elia M., Livesey G. (1983). Effects of ingested steak and infused leucine on forelimb metabolism in man and the fate of the carbon skeletons and amino groups of branched-chain amino acids. Clin. Sci..

[39] Suryawan A., Hawes J.W., Harris R.A., Shimomura Y., Jenkins A.E., Hutson S.M. (1998). A molecular model of human branched-chain amino acid metabolism. Am. J. Clin. Nutr..

[40] Tajiri K., Shimizu Y. (2013). Branched-chain amino acids in liver diseases. World J. Gastroenterol..

[41] D’Antona G., Ragni M., Cardile A., Tedesco L., Dossena M., Bruttini F., Caliaro F., Corsetti G., Bottinelli R., Carruba M.O. (2010). Branched-chain amino acid supplementation promotes survival and supports cardiac and skeletal muscle mitochondrial biogenesis in middle-aged mice. Cell Metabol..

[42] Strum J.C., Shehee R., Virley D., Richardson J., Mattie M., Selley P., Ghosh S., Nock C., Saunders A., Roses A. (2007). Rosiglitazone induces mitochondrial biogenesis in mouse brain. J. Alzheim. Dis..

[43] Tontonoz P., Hu E., Spiegelman B.M. (1994). Stimulation of adipogenesis in fibroblasts by ppar gamma 2, a lipid-activated transcription factor. Cell.

[44] Ichikawa K., Okabayashi T., Shima Y., Iiyama T., Takezaki Y., Munekage M., Namikawa T., Sugimoto T., Kobayashi M., Mimura T. (2012). Branched-chain amino acid-enriched nutrients stimulate antioxidant DNA repair in a rat model of liver injury induced by carbon tetrachloride. Mol. Biol. Rep..

[45] Blomstrand E., Eliasson J., Karlsson H.K., Kohnke R. (2006). Branched-chain amino acids activate key enzymes in protein synthesis after physical exercise. J. Nutr..

[46] Nishitani S., Matsumura T., Fujitani S., Sonaka I., Miura Y., Yagasaki K. (2002). Leucine promotes glucose uptake in skeletal muscles of rats. Biochem. Biophys. Res. Comm..

[47] Nishitani S., Takehana K., Fujitani S., Sonaka I. (2005). Branched-chain amino acids improve glucose metabolism in rats with liver cirrhosis. Am. J. Physiol. Gastrointest. Liver Physiol..

[48] Hinault C., Mothe-Satney I., Gautier N., Lawrence J.C., van Obberghen E. (2004). Amino acids and leucine allow insulin activation of the pkb/mtor pathway in normal adipocytes treated with wortmannin and in adipocytes from db/db mice. FASEB J..

[49] Miller W.L., Bose H.S. (2011). Early steps in steroidogenesis: Intracellular cholesterol trafficking. J. Lipid Res..

[50] Zhao S., Xu W., Jiang W., Yu W., Lin Y., Zhang T., Yao J., Zhou L., Zeng Y., Li H. (2010). Regulation of cellular metabolism by protein lysine acetylation. Science.

[51] Jiang W., Wang S., Xiao M., Lin Y., Zhou L., Lei Q., Xiong Y., Guan K.L., Zhao S. (2011). Acetylation regulates gluconeogenesis by promoting pepck1 degradation via recruiting the ubr5 ubiquitin ligase. Mol. Cell.

[52] Xiong Y., Guan K.L. (2012). Mechanistic insights into the regulation of metabolic enzymes by acetylation. J. Cell Biol..

[53] Brand M.D., Nicholls D.G. (2011). Assessing mitochondrial dysfunction in cells. Biochem. J..

[54] Tuppen H.A., Blakely E.L., Turnbull D.M., Taylor R.W. (2010). Mitochondrial DNA mutations and human disease. Biochim. Biophys. Acta.

[55] Park C.B., Larsson N.G. (2011). Mitochondrial DNA mutations in disease and aging. J. Cell Biol..

[56] Dudkina N.V., Kouril R., Peters K., Braun H.P., Boekema E.J. (2010). Structure and function of mitochondrial supercomplexes. Biochim. Biophys. Acta.

[57] Vannuvel K., Renard P., Raes M., Arnould T. (2013). Functional and morphological impact of er stress on mitochondria. J. Cell. Physiol..

[58] Pellegrino M.W., Nargund A.M., Haynes C.M. (2013). Signaling the mitochondrial unfolded protein response. Biochim. Biophys. Acta.

[59] Rousset S., Alves-Guerra M.C., Mozo J., Miroux B., Cassard-Doulcier A.M., Bouillaud F., Ricquier D. (2004). The biology of mitochondrial uncoupling proteins. Diabetes.

[60] Ricquier D., Bouillaud F., Toumelin P., Mory G., Bazin R., Arch J., Penicaud L. (1986). Expression of uncoupling protein mrna in thermogenic or weakly thermogenic brown adipose tissue. Evidence for a rapid beta-adrenoreceptor-mediated and transcriptionally regulated step during activation of thermogenesis. J. Biol. Chem..

[61] Levy S.E., Chen Y.S., Graham B.H., Wallace D.C. (2000). Expression and sequence analysis of the mouse adenine nucleotide translocase 1 and 2 genes. Gene.

[62] Stepien G., Torroni A., Chung A.B., Hodge J.A., Wallace D.C. (1992). Differential expression of adenine nucleotide translocator isoforms in mammalian tissues and during muscle cell differentiation. J. Biol.Chem..

[63] Brower J.V., Rodic N., Seki T., Jorgensen M., Fliess N., Yachnis A.T., McCarrey J.R., Oh S.P., Terada N. (2007). Evolutionarily conserved mammalian adenine nucleotide translocase 4 is essential for spermatogenesis. J. Biol. Chem..

[64] Brand M.D., Pakay J.L., Ocloo A., Kokoszka J., Wallace D.C., Brookes P.S., Cornwall E.J. (2005). The basal proton conductance of mitochondria depends on adenine nucleotide translocase content. Biochem. J..

[65] Liu Y., Chen X.J. (2013). Adenine nucleotide translocase, mitochondrial stress, and degenerative cell death. Oxidative Med. Cell. Longev..

[66] Liu J., Li J., Li W.J., Wang C.M. (2013). The role of uncoupling proteins in diabetes mellitus. J. Diabetes Res..

[67] Klingenberg M., Winkler E. (1985). The reconstituted isolated uncoupling protein is a membrane potential driven h+ translocator. EMBO J..

[68] Matthias A., Ohlson K.B., Fredriksson J.M., Jacobsson A., Nedergaard J., Cannon B. (2000). Thermogenic responses in brown fat cells are fully ucp1-dependent. Ucp2 or ucp3 do not substitute for ucp1 in adrenergically or fatty scid-induced thermogenesis. J. Biol. Chem..

[69] Cadrin M., Tolszczuk M., Guy J., Pelletier G., Freeman K.B., Bukowiecki L.J. (1985). Immunohistochemical identification of the uncoupling protein in rat brown adipose tissue. J. Histochem. Cytochem..

[70] Petrovic N., Walden T.B., Shabalina I.G., Timmons J.A., Cannon B., Nedergaard J. (2010). Chronic peroxisome proliferator-activated receptor gamma (ppargamma) activation of epididymally derived white adipocyte cultures reveals a population of thermogenically competent, ucp1-containing adipocytes molecularly distinct from classic brown adipocytes. J. Biol. Chem..

[71] Boss O., Samec S., Paoloni-Giacobino A., Rossier C., Dulloo A., Seydoux J., Muzzin P., Giacobino J.P. (1997). Uncoupling protein-3: A new member of the mitochondrial carrier family with tissue-specific expression. FEBS Lett..

[72] Sale M.M., Hsu F.C., Palmer N.D., Gordon C.J., Keene K.L., Borgerink H.M., Sharma A.J., Bergman R.N., Taylor K.D., Saad M.F. (2007). The uncoupling protein 1 gene, ucp1, is expressed in mammalian islet cells and associated with acute insulin response to glucose in african american families from the iras family study. BMC Endocr. Disord..

[73] Chen Y., Li Z.Y., Yang Y., Zhang H.J. (2012). Uncoupling protein 2 regulates glucagon-like peptide-1 secretion in l-cells. World J. Gastroenterol..

[74] Fleury C., Neverova M., Collins S., Raimbault S., Champigny O., Levi-Meyrueis C., Bouillaud F., Seldin M.F., Surwit R.S., Ricquier D. (1997). Uncoupling protein-2: A novel gene linked to obesity and hyperinsulinemia. Nat. Genet..

[75] Boss O., Samec S., Dulloo A., Seydoux J., Muzzin P., Giacobino J.P. (1997). Tissue-dependent upregulation of rat uncoupling protein-2 expression in response to fasting or cold. FEBS Lett..

[76] Li W., Nichols K., Nathan C.A., Zhao Y. (2013). Mitochondrial uncoupling protein 2 is up-regulated in human head and neck, skin, pancreatic, and prostate tumors. Canc. Biomarkers.

[77] Li Y., Maedler K., Shu L., Haataja L. (2008). Ucp-2 and ucp-3 proteins are differentially regulated in pancreatic beta-cells. PloS One.

[78] Mao W., Yu X.X., Zhong A., Li W., Brush J., Sherwood S.W., Adams S.H., Pan G. (1999). Ucp4, a novel brain-specific mitochondrial protein that reduces membrane potential in mammalian cells. FEBS Lett..

[79] Yu X.X., Mao W., Zhong A., Schow P., Brush J., Sherwood S.W., Adams S.H., Pan G. (2000). Characterization of novel ucp5/bmcp1 isoforms and differential regulation of ucp4 and ucp5 expression through dietary or temperature manipulation. FASEB J..

[80] Bouillaud F., Ricquier D., Thibault J., Weissenbach J. (1985). Molecular approach to thermogenesis in brown adipose tissue: Cdna cloning of the mitochondrial uncoupling protein. Proc. Natl. Acad. of Sci. USA.

[81] Kukat A., Dogan S.A., Edgar D., Mourier A., Jacoby C., Maiti P., Mauer J., Becker C., Senft K., Wibom R. (2014). Loss of ucp2 attenuates mitochondrial dysfunction without altering ros production and uncoupling activity. PLoS Genet..

[82] Adjeitey C.N., Mailloux R.J., Dekemp R.A., Harper M.E. (2013). Mitochondrial uncoupling in skeletal muscle by ucp1 augments energy expenditure and glutathione content while mitigating ros production. Am. J. Physiol. Endocrinol. Metabol..

[83] Shabalina I.G., Vrbacky M., Pecinova A., Kalinovich A.V., Drahota Z., Houstek J., Mracek T., Cannon B., Nedergaard J. (2014). Ros production in brown adipose tissue mitochondria: The question of ucp1-dependence. Biochim. Biophys. Acta.

[84] Shabalina I.G., Petrovic N., de Jong J.M., Kalinovich A.V., Cannon B., Nedergaard J. (2013). Ucp1 in brite/beige adipose tissue mitochondria is functionally thermogenic. Cell Rep..

[85] Wang Y., Huang L., Abdelrahim M., Cai Q., Truong A., Bick R., Poindexter B., Sheikh-Hamad D. (2009). Stanniocalcin-1 suppresses superoxide generation in macrophages through induction of mitochondrial ucp2. J. Leukocyte Biol..

[86] Tian X.Y., Wong W.T., Xu A., Lu Y., Zhang Y., Wang L., Cheang W.S., Wang Y., Yao X., Huang Y. (2012). Uncoupling protein-2 protects endothelial function in diet-induced obese mice. Circ. Res..

[87] Dando I., Fiorini C., Pozza E.D., Padroni C., Costanzo C., Palmieri M., Donadelli M. (2013). Ucp2 inhibition triggers ros-dependent nuclear translocation of gapdh and autophagic cell death in pancreatic adenocarcinoma cells. Biochim. Biophys. Acta.

[88] Vozza A., Parisi G., De Leonardis F., Lasorsa F.M., Castegna A., Amorese D., Marmo R., Calcagnile V.M., Palmieri L., Ricquier D. (2014). Ucp2 transports c4 metabolites out of mitochondria, regulating glucose and glutamine oxidation. Proc. Natl. Acad. Sci. USA.

[89] Clapham J.C., Arch J.R., Chapman H., Haynes A., Lister C., Moore G.B., Piercy V., Carter S.A., Lehner I., Smith S.A. (2000). Mice overexpressing human uncoupling protein-3 in skeletal muscle are hyperphagic and lean. Nature.

[90] Himms-Hagen J., Harper M.E. (2001). Physiological role of ucp3 may be export of fatty acids from mitochondria when fatty acid oxidation predominates: An hypothesis. Exp. Biol. Med..

[91] Trenker M., Malli R., Fertschai I., Levak-Frank S., Graier W.F. (2007). Uncoupling proteins 2 and 3 are fundamental for mitochondrial Ca^2+^ uniport. Nat. Cell Biol..

[92] Hoang T., Smith M.D., Jelokhani-Niaraki M. (2012). Toward understanding the mechanism of ion transport activity of neuronal uncoupling proteins ucp2, ucp4, and ucp5. Biochemistry.

[93] Gao C.L., Zhu J.G., Zhao Y.P., Chen X.H., Ji C.B., Zhang C.M., Zhu C., Xia Z.K., Peng Y.Z., Guo X.R. (2010). Mitochondrial dysfunction is induced by the overexpression of ucp4 in 3t3-l1 adipocytes. Int. J. Mol. Med..

[94] Ho J.W., Ho P.W., Zhang W.Y., Liu H.F., Kwok K.H., Yiu D.C., Chan K.H., Kung M.H., Ramsden D.B., Ho S.L. (2010). Transcriptional regulation of ucp4 by nf-kappab and its role in mediating protection against mpp+ toxicity. Free Radic. Biol. Med..

[95] Pfeiffer M., Kayzer E.B., Yang X., Abramson E., Kenaston M.A., Lago C.U., Lo H.H., Sedensky M.M., Lunceford A., Clarke C.F. (2011). Caenorhabditis elegans ucp4 protein controls complex ii-mediated oxidative phosphorylation through succinate transport. J. Biol. Chem..

[96] Fukai T., Ushio-Fukai M. (2011). Superoxide dismutases: Role in redox signaling, vascular function, and diseases. Antioxid. Redox Sign..

[97] Kirkman H.N., Gaetani G.F. (2007). Mammalian catalase: A venerable enzyme with new mysteries. Trends Biochem. Sci..

[98] Lu J., Holmgren A. (2014). The thioredoxin antioxidant system. Free Radic. Biol. Med..

[99] Wood Z.A., Schroder E., Robin Harris J., Poole L.B. (2003). Structure, mechanism and regulation of peroxiredoxins. Trends Biochem. Sci..

[100] Lundberg M., Johansson C., Chandra J., Enoksson M., Jacobsson G., Ljung J., Johansson M., Holmgren A. (2001). Cloning and expression of a novel human glutaredoxin (grx2) with mitochondrial and nuclear isoforms. J. Biol. Chem..

[101] Nogueiras R., Habegger K.M., Chaudhary N., Finan B., Banks A.S., Dietrich M.O., Horvath T.L., Sinclair D.A., Pfluger P.T., Tschop M.H. (2012). Sirtuin 1 and sirtuin 3: Physiological modulators of metabolism. Physiol. Rev..

[102] Cui Y., Xu X., Bi H., Zhu Q., Wu J., Xia X., Qiushi R., Ho P.C. (2006). Expression modification of uncoupling proteins and mnsod in retinal endothelial cells and pericytes induced by high glucose: The role of reactive oxygen species in diabetic retinopathy. Exp. Eye Res..

[103] Dymkowska D., Drabarek B., Podszywalow-Bartnicka P., Szczepanowska J., Zablocki K. (2014). Hyperglycaemia modifies energy metabolism and reactive oxygen species formation in endothelial cells *in vitro*. Arch. Biochem. Biophys..

[104] Koziel A., Woyda-Ploszczyca A., Kicinska A., Jarmuszkiewicz W. (2012). The influence of high glucose on the aerobic metabolism of endothelial ea.Hy926 cells. Pflugers Arch. Eur. J. Physiol..

[105] Matsuzaki H., Daitoku H., Hatta M., Aoyama H., Yoshimochi K., Fukamizu A. (2005). Acetylation of foxo1 alters its DNA-binding ability and sensitivity to phosphorylation. Proc. Natl. Acad. Sci. USA.

[106] Nakae J., Cao Y., Oki M., Orba Y., Sawa H., Kiyonari H., Iskandar K., Suga K., Lombes M., Hayashi Y. (2008). Forkhead transcription factor foxo1 in adipose tissue regulates energy storage and expenditure. Diabetes.

[107] Komelina N.P., Amerkhanov Z.G. (2010). A comparative study of the inhibitory effects of purine nucleotides and carboxyatractylate on the uncoupling protein-3 and adenine nucleotide translocase. Acta Biochim. Pol..

[108] Winkler E., Wachter E., Klingenberg M. (1997). Identification of the ph sensor for nucleotide binding in the uncoupling protein from brown adipose tissue. Biochemistry.

[109] Winkler E., Klingenberg M. (1992). Photoaffinity labeling of the nucleotide-binding site of the uncoupling protein from hamster brown adipose tissue. Eur. J. Biochem..

[110] Arechaga I., Ledesma A., Rial E. (2001). The mitochondrial uncoupling protein ucp1: A gated pore. IUBMB Life.

[111] Du Y., Meng Q., Zhang Q., Guo F. (2012). Isoleucine or valine deprivation stimulates fat loss via increasing energy expenditure and regulating lipid metabolism in wat. Amino Acids.

[112] Cheng Y., Meng Q., Wang C., Li H., Huang Z., Chen S., Xiao F., Guo F. (2010). Leucine deprivation decreases fat mass by stimulation of lipolysis in white adipose tissue and upregulation of uncoupling protein 1 (ucp1) in brown adipose tissue. Diabetes.

[113] Cheng Y., Zhang Q., Meng Q., Xia T., Huang Z., Wang C., Liu B., Chen S., Xiao F., Du Y. (2011). Leucine deprivation stimulates fat loss via increasing crh expression in the hypothalamus and activating the sympathetic nervous system. Mol. Endocrinol..

[114] Bernardi P., Penzo D., Wojtczak L. (2002). Mitochondrial energy dissipation by fatty acids. Mechanisms and implications for cell death. Vitam. Horm..

[115] Schonfeld P., Wojtczak L. (2007). Fatty acids decrease mitochondrial generation of reactive oxygen species at the reverse electron transport but increase it at the forward transport. Biochim. Biophys. Acta.

[116] Cole M.A., Murray A.J., Cochlin L.E., Heather L.C., McAleese S., Knight N.S., Sutton E., Jamil A.A., Parassol N., Clarke K. (2011). A high fat diet increases mitochondrial fatty acid oxidation and uncoupling to decrease efficiency in rat heart. Basic Res. Cardiol..

[117] Lambertucci R.H., Leandro C.G., Vinolo M.A., Nachbar R.T., Dos Reis Silveira L., Hirabara S.M., Curi R., Pithon-Curi T.C. (2012). The effects of palmitic acid on nitric oxide production by rat skeletal muscle: Mechanism via superoxide and inos activation. Cell. Physiol. Biochem.

[118] Divakaruni A.S., Humphrey D.M., Brand M.D. (2012). Fatty acids change the conformation of uncoupling protein 1 (ucp1). J. Biol. Chem..

[119] Fedorenko A., Lishko P.V., Kirichok Y. (2012). Mechanism of fatty-acid-dependent ucp1 uncoupling in brown fat mitochondria. Cell.

[120] Beck V., Jaburek M., Demina T., Rupprecht A., Porter R.K., Jezek P., Pohl E.E. (2007). Polyunsaturated fatty acids activate human uncoupling proteins 1 and 2 in planar lipid bilayers. FASEB J..

[121] Taltavull N., Munoz-Cortes M., Lluis L., Jove M., Fortuno A., Molinar-Toribio E., Torres J.L., Pazos M., Medina I., Nogues M.R. (2014). Eicosapentaenoic acid/docosahexaenoic acid 1:1 ratio improves histological alterations in obese rats with metabolic syndrome. Lipids Health Dis..

[122] Oster R.T., Tishinsky J.M., Yuan Z., Robinson L.E. (2010). Docosahexaenoic acid increases cellular adiponectin mrna and secreted adiponectin protein, as well as ppargamma mrna, in 3t3-l1 adipocytes. Appl. Physiol. Nutr. Metabol..

[123] Sadurskis A., Dicker A., Cannon B., Nedergaard J. (1995). Polyunsaturated fatty acids recruit brown adipose tissue: Increased ucp content and nst capacity. Am. J. Physiol..

[124] Jeckel K.M., Veeramachaneni D.N., Chicco A.J., Chapman P.L., Mulligan C.M., Hegarty J.R., Pagliassotti M.J., Ferguson L.A., Bouma G.J., Frye M.A. (2012). Docosahexaenoic acid supplementation does not improve western diet-induced cardiomyopathy in rats. PloS One.

[125] Wu Y., Zhang C., Dong Y., Wang S., Song P., Viollet B., Zou M.H. (2012). Activation of the amp-activated protein kinase by eicosapentaenoic acid (epa, 20:5 n-3) improves endothelial function *in vivo*. PloS One.

[126] Flachs P., Horakova O., Brauner P., Rossmeisl M., Pecina P., Franssen-van Hal N., Ruzickova J., Sponarova J., Drahota Z., Vlcek C. (2005). Polyunsaturated fatty acids of marine origin upregulate mitochondrial biogenesis and induce beta-oxidation in white fat. Diabetologia.

[127] Janovska P., Flachs P., Kazdova L., Kopecky J. (2013). Anti-obesity effect of n-3 polyunsaturated fatty acids in mice fed high-fat diet is independent of cold-induced thermogenesis. Physiol.Res..

[128] Flachs P., Ruhl R., Hensler M., Janovska P., Zouhar P., Kus V., Macek Jilkova Z., Papp E., Kuda O., Svobodova M. (2011). Synergistic induction of lipid catabolism and anti-inflammatory lipids in white fat of dietary obese mice in response to calorie restriction and n-3 fatty acids. Diabetologia.

[129] Flachs P., Rossmeisl M., Bryhn M., Kopecky J. (2009). Cellular and molecular effects of n-3 polyunsaturated fatty acids on adipose tissue biology and metabolism. Clin. Sci..

[130] Griffiths D.E., Cain K., Hyams R.L. (1977). Studies of energy-linked reactions. Inhibition of oxidative phosphorylation by dl-8-methyldihydrolipoate. Biochem. J..

[131] Valdecantos M.P., Perez-Matute P., Gonzalez-Muniesa P., Prieto-Hontoria P.L., Moreno-Aliaga M.J., Martinez J.A. (2012). Lipoic acid administration prevents nonalcoholic steatosis linked to long-term high-fat feeding by modulating mitochondrial function. J. Nutr. Biochem..

[132] Wang Y., Li X., Guo Y., Chan L., Guan X. (2010). Alpha-lipoic acid increases energy expenditure by enhancing adenosine monophosphate-activated protein kinase-peroxisome proliferator-activated receptor-gamma coactivator-1alpha signaling in the skeletal muscle of aged mice. Metabol. Clin. Exp..

[133] Khamaisi M., Potashnik R., Tirosh A., Demshchak E., Rudich A., Tritschler H., Wessel K., Bashan N. (1997). Lipoic acid reduces glycemia and increases muscle glut4 content in streptozotocin-diabetic rats. Metabol. Clin. Exp..

[134] Rudich A., Tirosh A., Potashnik R., Khamaisi M., Bashan N. (1999). Lipoic acid protects against oxidative stress induced impairment in insulin stimulation of protein kinase b and glucose transport in 3t3-l1 adipocytes. Diabetologia.

[135] Valdecantos M.P., Perez-Matute P., Quintero P., Martinez J.A. (2010). Vitamin c, resveratrol and lipoic acid actions on isolated rat liver mitochondria: All antioxidants but different. Redox Rep..

[136] Tonin A.M., Amaral A.U., Busanello E.N., Grings M., Castilho R.F., Wajner M. (2013). Long-chain 3-hydroxy fatty acids accumulating in long-chain 3-hydroxyacyl-coa dehydrogenase and mitochondrial trifunctional protein deficiencies uncouple oxidative phosphorylation in heart mitochondria. J. Bioenerg. Biomembr..

[137] Sarkar P., Zaja I., Bienengraeber M., Rarick K.R., Terashvili M., Canfield S., Falck J.R., Harder D.R. (2013). Epoxyeicosatrienoic acids pre-treatment improves amyloid beta-induced mitochondrial dysfunction in cultured rat hippocampal astrocytes. Am. J. Physiol. Heart Circ. Physiol..

[138] Echtay K.S., Esteves T.C., Pakay J.L., Jekabsons M.B., Lambert A.J., Portero-Otin M., Pamplona R., Vidal-Puig A.J., Wang S., Roebuck S.J. (2003). A signalling role for 4-hydroxy-2-nonenal in regulation of mitochondrial uncoupling. EMBO J..

[139] Malingriaux E.A., Rupprecht A., Gille L., Jovanovic O., Jezek P., Jaburek M., Pohl E.E. (2013). Fatty acids are key in 4-hydroxy-2-nonenal-mediated activation of uncoupling proteins 1 and 2. PloS One.

[140] Nadtochiy S.M., Zhu Q., Urciuoli W., Rafikov R., Black S.M., Brookes P.S. (2012). Nitroalkenes confer acute cardioprotection via adenine nucleotide translocase 1. J. Biol. Chem..

[141] Senese R., Valli V., Moreno M., Lombardi A., Busiello R.A., Cioffi F., Silvestri E., Goglia F., Lanni A., de Lange P. (2011). Uncoupling protein 3 expression levels influence insulin sensitivity, fatty acid oxidation, and related signaling pathways. Pflug. Arch..

[142] Srivastava S., Kashiwaya Y., King M.T., Baxa U., Tam J., Niu G., Chen X., Clarke K., Veech R.L. (2012). Mitochondrial biogenesis and increased uncoupling protein 1 in brown adipose tissue of mice fed a ketone ester diet. FASEB J..

[143] Kashiwaya Y., Pawlosky R., Markis W., King M.T., Bergman C., Srivastava S., Murray A., Clarke K., Veech R.L. (2010). A ketone ester diet increases brain malonyl-coa and uncoupling proteins 4 and 5 while decreasing food intake in the normal wistar rat. J. Biol. Chem..

[144] Sullivan P.G., Rippy N.A., Dorenbos K., Concepcion R.C., Agarwal A.K., Rho J.M. (2004). The ketogenic diet increases mitochondrial uncoupling protein levels and activity. Ann. Neurol..

[145] Jikumaru M., Hiramoto K., Honma T., Sato E.F., Sekiyama A., Inoue M. (2007). Effect of starvation on the survival of male and female mice. Physiol. Chem. Phys. Med. NMR.

[146] Sanchez-Alvarez R., Martinez-Outschoorn U.E., Lamb R., Hulit J., Howell A., Gandara R., Sartini M., Rubin E., Lisanti M.P., Sotgia F. (2013). Mitochondrial dysfunction in breast cancer cells prevents tumor growth: Understanding chemoprevention with metformin. Cell Cycle.

[147] Mercader J., Ribot J., Murano I., Felipe F., Cinti S., Bonet M.L., Palou A. (2006). Remodeling of white adipose tissue after retinoic acid administration in mice. Endocrinology.

[148] Amengual J., Ribot J., Bonet M.L., Palou A. (2010). Retinoic acid treatment enhances lipid oxidation and inhibits lipid biosynthesis capacities in the liver of mice. Cell. Physiol. Biochem..

[149] Camara Y., Mampel T., Armengol J., Villarroya F., Dejean L. (2009). Ucp3 expression in liver modulates gene expression and oxidative metabolism in response to fatty acids, and sensitizes mitochondria to permeability transition. Cell. Phys. Biochem..

[150] Pietta P.G. (2000). Flavonoids as antioxidants. J. Nat. Prod..

[151] Dorta D.J., Pigoso A.A., Mingatto F.E., Rodrigues T., Pestana C.R., Uyemura S.A., Santos A.C., Curti C. (2008). Antioxidant activity of flavonoids in isolated mitochondria. Phytother. Res..

[152] Miyashita K., Nishikawa S., Beppu F., Tsukui T., Abe M., Hosokawa M. (2011). The allenic carotenoid fucoxanthin, a novel marine nutraceutical from brown seaweeds. J. Sci. Food Agr..

[153] Schuster S., Fell D.A., Dandekar T. (2000). A general definition of metabolic pathways useful for systematic organization and analysis of complex metabolic networks. Nat. Biotechnol..

[154] Edwards J.S., Ibarra R.U., Palsson B.O. (2001). In silico predictions of escherichia coli metabolic capabilities are consistent with experimental data. Nat. Biotechnol..

[155] Gowda G.A., Zhang S., Gu H., Asiago V., Shanaiah N., Raftery D. (2008). Metabolomics-based methods for early disease diagnostics. Expert Rev. Mol. Diagn..

[156] Shulaev V. (2006). Metabolomics technology and bioinformatics. Brief. Bioinform..

[157] Beecher C.W.W., Harrigan G.G., Goodacre R (2003). The human metabolome. Metabolic Profiling: Its Role in Biomarker Discovery and Gene Function Analysis.

[158] Wishart D.S., Tzur D., Knox C., Eisner R., Guo A.C., Young N., Cheng D., Jewell K., Arndt D., Sawhney S. (2007). Hmdb: The human metabolome database. Nucleic Acids Res..

[159] Weckwerth W., Wenzel K., Fiehn O. (2004). Process for the integrated extraction, identification and quantification of metabolites, proteins and rna to reveal their co-regulation in biochemical networks. Proteomics.

[160] Fan T.W., Lorkiewicz P.K., Sellers K., Moseley H.N., Higashi R.M., Lane A.N. (2012). Stable isotope-resolved metabolomics and applications for drug development. Pharmacol. Therapeut..

[161] Smolinska A., Blanchet L., Buydens L.M., Wijmenga S.S. (2012). Nmr and pattern recognition methods in metabolomics: From data acquisition to biomarker discovery: A review. Anal. Chim. Acta.

[162] Zhou B., Xiao J.F., Tuli L., Ressom H.W. (2012). Lc-ms-based metabolomics. Mol. Biosyst..

[163] Koek M.M., Jellema R.H., van der Greef J., Tas A.C., Hankemeier T. (2011). Quantitative metabolomics based on gas chromatography mass spectrometry: Status and perspectives. Metabolomics.

[164] Werner E., Heilier J.F., Ducruix C., Ezan E., Junot C., Tabet J.C. (2008). Mass spectrometry for the identification of the discriminating signals from metabolomics: Current status and future trends. J. Chromatogr. B.

[165] Bakken I.J., Sonnewald U., Clark J.B., Bates T.E. (1997). [U-13C]glutamate metabolism in rat brain mitochondria reveals malic enzyme activity. Neuroreport.

[166] Teng Q., Ekman D.R., Huang W., Collette T.W. (2012). Push-through direct injection nmr: An optimized automation method applied to metabolomics. Analyst.

[167] Matuszewski B.K., Constanzer M.L., Chavez-Eng C.M. (1998). Matrix effect in quantitative lc/ms/ms analyses of biological fluids: A method for determination of finasteride in human plasma at picogram per milliliter concentrations. Anal. Chem..

[168] Larger P.J., Breda M., Fraier D., Hughes H., James C.A. (2005). Ion-suppression effects in liquid chromatography-tandem mass spectrometry due to a formulation agent, a case study in drug discovery bioanalysis. J. Pharmaceut. Biomed. Anal..

[169] Lin Z., Vicente Goncalves C.M., Dai L., Lu H.M., Huang J.H., Ji H., Wang D.S., Yi L.Z., Liang Y.Z. (2014). Exploring metabolic syndrome serum profiling based on gas chromatography mass spectrometry and random forest models. Anal. Chim. Acta.

[170] Chang K.L., New L.S., Mal M., Goh C.W., Aw C.C., Browne E.R., Chan E.C. (2011). Metabolic profiling of 3-nitropropionic acid early-stage huntington’s disease rat model using gas chromatography time-of-flight mass spectrometry. J. Proteome Res..

[171] Halket J.M., Waterman D., Przyborowska A.M., Patel R.K., Fraser P.D., Bramley P.M. (2005). Chemical derivatization and mass spectral libraries in metabolic profiling by GC/MS and LC/MS/MS. J. Exp.Bot..

[172] Oberacher H., Whitley G., Berger B. (2013). Evaluation of the sensitivity of the “wiley registry of tandem mass spectral data, MSforID” with MS/MS data of the “NIST/NIH/EPA mass spectral library”. J. Mass Spectrom..

[173] Horai H., Arita M., Kanaya S., Nihei Y., Ikeda T., Suwa K., Ojima Y., Tanaka K., Tanaka S., Aoshima K. (2010). Massbank: A public repository for sharing mass spectral data for life sciences. J. Mass Spectrom..

[174] Heinonen M., Shen H., Zamboni N., Rousu J. (2012). Metabolite identification and molecular fingerprint prediction through machine learning. Bioinformatics.

[175] Gravel S.P., Andrzejewski S., Avizonis D., St-Pierre J. (2014). Stable isotope tracer analysis in isolated mitochondria from mammalian systems. Metabolites.

[176] Soga T. (2007). Capillary electrophoresis-mass spectrometry for metabolomics. Meth. Mol. Biol..

[177] World Health Organization (WHO) Obesity. http://www.who.int/topics/obesity/en/.

[178] Wronska A., Kmiec Z. (2012). Structural and biochemical characteristics of various white adipose tissue depots. Acta Physiol..

[179] Duncan R.E., Ahmadian M., Jaworski K., Sarkadi-Nagy E., Sul H.S. (2007). Regulation of lipolysis in adipocytes. Ann. Rev. Nutr..

[180] Lehr S., Hartwig S., Lamers D., Famulla S., Muller S., Hanisch F.G., Cuvelier C., Ruige J., Eckardt K., Ouwens D.M. (2012). Identification and validation of novel adipokines released from primary human adipocytes. Mol. Cell. Proteom..

[181] Trayhurn P., Wood I.S. (2004). Adipokines: Inflammation and the pleiotropic role of white adipose tissue. Br. J. Nutr..

[182] Molina H., Yang Y., Ruch T., Kim J.W., Mortensen P., Otto T., Nalli A., Tang Q.Q., Lane M.D., Chaerkady R. (2009). Temporal profiling of the adipocyte proteome during differentiation using a five-plex silac based strategy. J. Proteome Res..

[183] Zhong J., Krawczyk S.A., Chaerkady R., Huang H., Goel R., Bader J.S., Wong G.W., Corkey B.E., Pandey A. (2010). Temporal profiling of the secretome during adipogenesis in humans. J. Proteome Res..

[184] Alvarez-Llamas G., Szalowska E., de Vries M.P., Weening D., Landman K., Hoek A., Wolffenbuttel B.H., Roelofsen H., Vonk R.J. (2007). Characterization of the human visceral adipose tissue secretome. Mol. Cell. Proteom..

[185] Ouchi N., Parker J.L., Lugus J.J., Walsh K. (2011). Adipokines in inflammation and metabolic disease. Nat. Rev. Immunol..

[186] Bluher M. (2014). Adipokines—Removing road blocks to obesity and diabetes therapy. Mol. Metabol..

[187] Seale P., Bjork B., Yang W., Kajimura S., Chin S., Kuang S., Scime A., Devarakonda S., Conroe H.M., Erdjument-Bromage H. (2008). Prdm16 controls a brown fat/skeletal muscle switch. Nature.

[188] Tang Q.Q., Lane M.D. (2012). Adipogenesis: From stem cell to adipocyte. Ann. Rev. Biochem..

[189] Carobbio S., Rosen B., Vidal-Puig A. (2013). Adipogenesis: New insights into brown adipose tissue differentiation. J. Mol. Endocrinol..

[190] Heaton J.M. (1972). The distribution of brown adipose tissue in the human. J. Anat..

[191] Saito M., Okamatsu-Ogura Y., Matsushita M., Watanabe K., Yoneshiro T., Nio-Kobayashi J., Iwanaga T., Miyagawa M., Kameya T., Nakada K. (2009). High incidence of metabolically active brown adipose tissue in healthy adult humans: Effects of cold exposure and adiposity. Diabetes.

[192] Van der Lans A.A., Hoeks J., Brans B., Vijgen G.H., Visser M.G., Vosselman M.J., Hansen J., Jorgensen J.A., Wu J., Mottaghy F.M. (2013). Cold acclimation recruits human brown fat and increases nonshivering thermogenesis. J. Clin. Investig..

[193] Rosenwald M., Wolfrum C. (2014). The origin and definition of brite *versus* white and classical brown adipocytes. Adipocyte.

[194] Virtue S., Vidal-Puig A. (2013). Adipose tissue expandability, lipotoxicity and the metabolic syndrome. Biochim. Biophys. Acta.

[195] Huang P.L. (2009). A comprehensive definition for metabolic syndrome. Dis. Models Mech..

[196] Marchesini G., Moscatiello S., Di Domizio S., Forlani G. (2008). Obesity-associated liver disease. J. Clin. Endocrinol. Metabol..

[197] Lavie C.J., Milani R.V., Ventura H.O. (2009). Obesity and cardiovascular disease: Risk factor, paradox, and impact of weight loss. J. Am. Coll. Cardiol..

[198] Ligibel J. (2011). Obesity and breast cancer. Oncology.

[199] James A.M., Collins Y., Logan A., Murphy M.P. (2012). Mitochondrial oxidative stress and the metabolic syndrome. Trends Endocrinol. Metabol..

[200] Furukawa S., Fujita T., Shimabukuro M., Iwaki M., Yamada Y., Nakajima Y., Nakayama O., Makishima M., Matsuda M., Shimomura I. (2004). Increased oxidative stress in obesity and its impact on metabolic syndrome. J. Clin. Investig..

[201] Pereira S., Park E., Mori Y., Haber C.A., Han P., Uchida T., Stavar L., Oprescu A.I., Koulajian K., Ivovic A. (2014). Ffa-induced hepatic insulin resistance *in vivo* is mediated by pkc-delta, nadph oxidase, and oxidative stress. Am. J. Physiol. Endocrinol. Metabol..

[202] Hirao K., Maruyama T., Ohno Y., Hirose H., Shimada A., Takei I., Murata M., Morii T., Eguchi T., Hayashi M. (2010). Association of increased reactive oxygen species production with abdominal obesity in type 2 diabetes. Obes. Res. Clin. Pract..

[203] Degasperi G.R., Denis R.G., Morari J., Solon C., Geloneze B., Stabe C., Pareja J.C., Vercesi A.E., Velloso L.A. (2009). Reactive oxygen species production is increased in the peripheral blood monocytes of obese patients. Metabolism.

[204] Lefort N., Glancy B., Bowen B., Willis W.T., Bailowitz Z., De Filippis E.A., Brophy C., Meyer C., Hojlund K., Yi Z. (2010). Increased reactive oxygen species production and lower abundance of complex i subunits and carnitine palmitoyltransferase 1b protein despite normal mitochondrial respiration in insulin-resistant human skeletal muscle. Diabetes.

[205] Dandona P., Mohanty P., Hamouda W., Ghanim H., Aljada A., Garg R., Kumar V. (2001). Inhibitory effect of a two day fast on reactive oxygen species (ros) generation by leucocytes and plasma ortho-tyrosine and meta-tyrosine concentrations. J. Clin. Endocrinol. Metabol..

[206] Yu T., Robotham J.L., Yoon Y. (2006). Increased production of reactive oxygen species in hyperglycemic conditions requires dynamic change of mitochondrial morphology. Proc. Natl. Acad. Sci. USA.

[207] Ritov V.B., Menshikova E.V., He J., Ferrell R.E., Goodpaster B.H., Kelley D.E. (2005). Deficiency of subsarcolemmal mitochondria in obesity and type 2 diabetes. Diabetes.

[208] Rong J.X., Qiu Y., Hansen M.K., Zhu L., Zhang V., Xie M., Okamoto Y., Mattie M.D., Higashiyama H., Asano S. (2007). Adipose mitochondrial biogenesis is suppressed in db/db and high-fat diet-fed mice and improved by rosiglitazone. Diabetes.

[209] Wilson-Fritch L., Nicoloro S., Chouinard M., Lazar M.A., Chui P.C., Leszyk J., Straubhaar J., Czech M.P., Corvera S. (2004). Mitochondrial remodeling in adipose tissue associated with obesity and treatment with rosiglitazone. J. Clin. Investig..

[210] Lee J.Y., Lee D.C., Im J.A., Lee J.W. (2014). Mitochondrial DNA copy number in peripheral blood is independently associated with visceral fat accumulation in healthy young adults. Int. J. Endocrinol..

[211] Niemann B., Chen Y., Teschner M., Li L., Silber R.E., Rohrbach S. (2011). Obesity induces signs of premature cardiac aging in younger patients: The role of mitochondria. J. Am. Coll. Cardiol..

[212] Valerio A., Cardile A., Cozzi V., Bracale R., Tedesco L., Pisconti A., Palomba L., Cantoni O., Clementi E., Moncada S. (2006). Tnf-alpha downregulates enos expression and mitochondrial biogenesis in fat and muscle of obese rodents. J. Clin. Investig..

[213] Zorzano A., Hernandez-Alvarez M.I., Palacin M., Mingrone G. (2010). Alterations in the mitochondrial regulatory pathways constituted by the nuclear co-factors pgc-1alpha or pgc-1beta and mitofusin 2 in skeletal muscle in type 2 diabetes. Biochim. Biophys. Acta.

[214] Hakansson J., Eliasson B., Smith U., Enerback S. (2011). Adipocyte mitochondrial genes and the forkhead factor foxc2 are decreased in type 2 diabetes patients and normalized in response to rosiglitazone. Diabetol. Metab. Syndr..

[215] Austin S., St-Pierre J. (2012). Pgc1alpha and mitochondrial metabolism—Emerging concepts and relevance in ageing and neurodegenerative disorders. J. Cell Sci..

[216] Sharma K., Karl B., Mathew A.V., Gangoiti J.A., Wassel C.L., Saito R., Pu M., Sharma S., You Y.H., Wang L. (2013). Metabolomics reveals signature of mitochondrial dysfunction in diabetic kidney disease. J. Am. Soc. Nephrol..

[217] Fonseca V., Rosenstock J., Patwardhan R., Salzman A. (2000). Effect of metformin and rosiglitazone combination therapy in patients with type 2 diabetes mellitus: A randomized controlled trial. J. Am. Med. Assoc..

[218] Chanseaume E., Barquissau V., Salles J., Aucouturier J., Patrac V., Giraudet C., Gryson C., Duche P., Boirie Y., Chardigny J.M. (2010). Muscle mitochondrial oxidative phosphorylation activity, but not content, is altered with abdominal obesity in sedentary men: Synergism with changes in insulin sensitivity. J. Clin. Endocrinol. Metabol..

[219] Shen X., Zheng S., Thongboonkerd V., Xu M., Pierce W.M., Klein J.B., Epstein P.N. (2004). Cardiac mitochondrial damage and biogenesis in a chronic model of type 1 diabetes. Am. J. Physiol. Endocrinol. Metab..

[220] Kim J.A., Wei Y., Sowers J.R. (2008). Role of mitochondrial dysfunction in insulin resistance. Circ. Res..

[221] Petersen P. (1977). Abnormal mitochondria in hepatocytes in human fatty liver. Acta Pathol. Microbiol. Scand..

[222] Eura Y., Ishihara N., Yokota S., Mihara K. (2003). Two mitofusin proteins, mammalian homologues of fzo, with distinct functions are both required for mitochondrial fusion. J. Biochem..

[223] Bach D., Pich S., Soriano F.X., Vega N., Baumgartner B., Oriola J., Daugaard J.R., Lloberas J., Camps M., Zierath J.R. (2003). Mitofusin-2 determines mitochondrial network architecture and mitochondrial metabolism. A novel regulatory mechanism altered in obesity. J. Biol. Chem..

[224] Liu R., Jin P., LiqunYu, Wang Y., Han L., Shi T., Li X. (2014). Impaired mitochondrial dynamics and bioenergetics in diabetic skeletal muscle. PloS One.

[225] Hackenbrock C.R. (1968). Chemical and physical fixation of isolated mitochondria in low-energy and high-energy states. Proc. Natl. Acad. Sci. USA.

[226] Mogensen M., Sahlin K., Fernstrom M., Glintborg D., Vind B.F., Beck-Nielsen H., Hojlund K. (2007). Mitochondrial respiration is decreased in skeletal muscle of patients with type 2 diabetes. Diabetes.

[227] Martin S.D., Morrison S., Konstantopoulos N., McGee S.L. (2014). Mitochondrial dysfunction has divergent, cell type-dependent effects on insulin action. Mol. Metab..

[228] Sanz M.N., Sanchez-Martin C., Detaille D., Vial G., Rigoulet M., El-Mir M.Y., Rodriguez-Villanueva G. (2011). Acute mitochondrial actions of glitazones on the liver: A crucial parameter for their antidiabetic properties. Cell. Physiol. Biochem..

[229] Brunmair B., Staniek K., Gras F., Scharf N., Althaym A., Clara R., Roden M., Gnaiger E., Nohl H., Waldhausl W. (2004). Thiazolidinediones, like metformin, inhibit respiratory complex i: A common mechanism contributing to their antidiabetic actions?. Diabetes.

[230] Pagel-Langenickel I., Schwartz D.R., Arena R.A., Minerbi D.C., Johnson D.T., Waclawiw M.A., Cannon R.O., Balaban R.S., Tripodi D.J., Sack M.N. (2007). A discordance in rosiglitazone mediated insulin sensitization and skeletal muscle mitochondrial content/activity in type 2 diabetes mellitus. Am. J. Physiol. Heart Circ. Physiol..

[231] Newgard C.B., An J., Bain J.R., Muehlbauer M.J., Stevens R.D., Lien L.F., Haqq A.M., Shah S.H., Arlotto M., Slentz C.A. (2009). A branched-chain amino acid-related metabolic signature that differentiates obese and lean humans and contributes to insulin resistance. Cell Metabol..

[232] Prentice K.J., Luu L., Allister E.M., Liu Y., Jun L.S., Sloop K.W., Hardy A.B., Wei L., Jia W., Fantus I.G. (2014). The furan fatty acid metabolite cmpf is elevated in diabetes and induces beta cell dysfunction. Cell Metabol..

[233] Sampey B.P., Freemerman A.J., Zhang J., Kuan P.F., Galanko J.A., O’Connell T.M., Ilkayeva O.R., Muehlbauer M.J., Stevens R.D., Newgard C.B. (2012). Metabolomic profiling reveals mitochondrial-derived lipid biomarkers that drive obesity-associated inflammation. PloS One.

[234] Cummins T.D., Holden C.R., Sansbury B.E., Gibb A.A., Shah J., Zafar N., Tang Y., Hellmann J., Rai S.N., Spite M. (2014). Metabolic remodeling of white adipose tissue in obesity. Am. J. Physiol. Endocrinol. Metabol..

[235] Won E.Y., Yoon M.K., Kim S.W., Jung Y., Bae H.W., Lee D., Park S.G., Lee C.H., Hwang G.S., Chi S.W. (2013). Gender-specific metabolomic profiling of obesity in leptin-deficient ob/ob mice by 1h nmr spectroscopy. PloS One.

[236] Dudzik D., Zorawski M., Skotnicki M., Zarzycki W., Kozlowska G., Bibik-Malinowska K., Vallejo M., Garcia A., Barbas C., Ramos M.P. (2014). Metabolic fingerprint of gestational diabetes mellitus. J. Proteomics.

[237] Eisinger K., Krautbauer S., Hebel T., Schmitz G., Aslanidis C., Liebisch G., Buechler C. (2014). Lipidomic analysis of the liver from high-fat diet induced obese mice identifies changes in multiple lipid classes. Exp. Mol. Pathol..

[238] Reinehr T., Wolters B., Knop C., Lass N., Hellmuth C., Harder U., Peissner W., Wahl S., Grallert H., Adamski J. Changes in the serum metabolite profile in obese children with weight loss. Eur. J. Nutr..

[239] Schafer N., Yu Z., Wagener A., Millrose M.K., Reissmann M., Bortfeldt R., Dieterich C., Adamski J., Wang-Sattler R., Illig T. (2014). Changes in metabolite profiles caused by genetically determined obesity in mice. Metabolomics.

[240] Li F., Jiang C., Larsen M.C., Bushkofsky J., Krausz K.W., Wang T., Jefcoate C.R., Gonzalez F.J. (2014). Lipidomics reveals a link between cyp1b1 and scd1 in promoting obesity. J. Proteome Res..

[241] Suganami T., Tanaka M., Ogawa Y. (2012). Adipose tissue inflammation and ectopic lipid accumulation. Endocr. J..

[242] Nobuhara M., Saotome M., Watanabe T., Urushida T., Katoh H., Satoh H., Funaki M., Hayashi H. (2013). Mitochondrial dysfunction caused by saturated fatty acid loading induces myocardial insulin-resistance in differentiated h9c2 myocytes: A novel *ex vivo* myocardial insulin-resistance model. Exp. Cell Res..

[243] Yang C., Aye C.C., Li X., Diaz Ramos A., Zorzano A., Mora S. (2012). Mitochondrial dysfunction in insulin resistance: Differential contributions of chronic insulin and saturated fatty acid exposure in muscle cells. Biosci. Rep..

[244] Koves T.R., Ussher J.R., Noland R.C., Slentz D., Mosedale M., Ilkayeva O., Bain J., Stevens R., Dyck J.R., Newgard C.B. (2008). Mitochondrial overload and incomplete fatty acid oxidation contribute to skeletal muscle insulin resistance. Cell Metabol..

[245] Sevastou I., Kaffe E., Mouratis M.A., Aidinis V. (2013). Lysoglycerophospholipids in chronic inflammatory disorders: The PLA(2)/LPC and ATX/LPA axes. Biochim. Biophys. Acta.

[246] Moolenaar W.H., Hla T. (2012). Snapshot: Bioactive lysophospholipids. Cell.

[247] Basanez G., Sharpe J.C., Galanis J., Brandt T.B., Hardwick J.M., Zimmerberg J. (2002). Bax-type apoptotic proteins porate pure lipid bilayers through a mechanism sensitive to intrinsic monolayer curvature. J. Biol. Chem..

[248] Kalous M., Rauchova H., Drahota Z. (1992). The effect of lysophosphatidylcholine on the activity of various mitochondrial enzymes. Biochim. Biophys. Acta.

[249] Kakisaka K., Cazanave S.C., Fingas C.D., Guicciardi M.E., Bronk S.F., Werneburg N.W., Mott J.L., Gores G.J. (2012). Mechanisms of lysophosphatidylcholine-induced hepatocyte lipoapoptosis. Am. J. Physiol..

[250] Eisinger K., Liebisch G., Schmitz G., Aslanidis C., Krautbauer S., Buechler C. (2014). Lipidomic analysis of serum from high fat diet induced obese mice. Int. J. Mol. Sci..

[251] Nestel P.J., Straznicky N., Mellett N.A., Wong G., de Souza D.P., Tull D.L., Barlow C.K., Grima M.T., Meikle P.J. (2014). Specific plasma lipid classes and phospholipid fatty acids indicative of dairy food consumption associate with insulin sensitivity. Am. J. Clin. Nutr..

[252] Watkins S.M., Reifsnyder P.R., Pan H.J., German J.B., Leiter E.H. (2002). Lipid metabolome-wide effects of the ppargamma agonist rosiglitazone. J. Lipid Res..

[253] Bao Y., Zhao T., Wang X., Qiu Y., Su M., Jia W. (2009). Metabonomic variations in the drug-treated type 2 diabetes mellitus patients and healthy volunteers. J. Proteome Res..

[254] Zhu Y., Feng Y., Shen L., Xu D., Wang B., Ruan K., Cong W. (2013). Effect of metformin on the urinary metabolites of diet-induced-obese mice studied by ultra performance liquid chromatography coupled to time-of-flight mass spectrometry (UPLC-TOF/MS). J. Chromatogr. B.

[255] Huo T., Cai S., Lu X., Sha Y., Yu M., Li F. (2009). Metabonomic study of biochemical changes in the serum of type 2 diabetes mellitus patients after the treatment of metformin hydrochloride. J. Pharmaceut. Biomed. Anal..

[256] Simon-Szabo L., Kokas M., Mandl J., Keri G., Csala M. (2014). Metformin attenuates palmitate-induced endoplasmic reticulum stress, serine phosphorylation of irs-1 and apoptosis in rat insulinoma cells. PloS One.

[257] Clarke C., Xiao R., Place E., Zhang Z., Sondheimer N., Bennett M., Yudkoff M., Falk M.J. (2013). Mitochondrial respiratory chain disease discrimination by retrospective cohort analysis of blood metabolites. Mol. Genet. Metab..

[258] Eaton S. (2002). Control of mitochondrial beta-oxidation flux. Prog. Lipid Res..

[259] Shah S.H., Crosslin D.R., Haynes C.S., Nelson S., Turer C.B., Stevens R.D., Muehlbauer M.J., Wenner B.R., Bain J.R., Laferrere B. (2012). Branched-chain amino acid levels are associated with improvement in insulin resistance with weight loss. Diabetologia.

[260] She P., Reid T.M., Bronson S.K., Vary T.C., Hajnal A., Lynch C.J., Hutson S.M. (2007). Disruption of bcatm in mice leads to increased energy expenditure associated with the activation of a futile protein turnover cycle. Cell Metab..

[261] Walford G.A., Davis J., Warner A.S., Ackerman R.J., Billings L.K., Chamarthi B., Fanelli R.R., Hernandez A.M., Huang C., Khan S.Q. (2013). Branched chain and aromatic amino acids change acutely following two medical therapies for type 2 diabetes mellitus. Metabolism.

[262] Hsiao G., Chapman J., Ofrecio J.M., Wilkes J., Resnik J.L., Thapar D., Subramaniam S., Sears D.D. (2011). Multi-tissue, selective ppargamma modulation of insulin sensitivity and metabolic pathways in obese rats. Am. J. Physiol. Endocrinol. Metab..

[263] Yang R., Dong J., Zhao H., Li H., Guo H., Wang S., Zhang C., Wang S., Wang M., Yu S. (2014). Association of branched-chain amino acids with carotid intima-media thickness and coronary artery disease risk factors. PloS One.

[264] Seifert E.L., Fiehn O., Bezaire V., Bickel D.R., Wohlgemuth G., Adams S.H., Harper M.E. (2010). Long-chain fatty acid combustion rate is associated with unique metabolite profiles in skeletal muscle mitochondria. PloS One.

[265] American Diabetes Association (2010). Diagnosis and classification of diabetes mellitus. Diabetes Care.

[266] Gohring I., Sharoyko V.V., Malmgren S., Andersson L.E., Spegel P., Nicholls D.G., Mulder H. (2014). Chronic high glucose and pyruvate levels differentially affect mitochondrial bioenergetics and fuel-stimulated insulin secretion from clonal ins-1 832/13 cells. J. Biol. Chem..

[267] Janzer A., German N.J., Gonzalez-Herrera K.N., Asara J.M., Haigis M.C., Struhl K. (2014). Metformin and phenformin deplete tricarboxylic acid cycle and glycolytic intermediates during cell transformation and ntps in cancer stem cells. Proc. Natl. Acad. Sci. USA.

[268] Jang E.H., Kim H.K., Park C.S., Kang J.H. (2010). Increased expression of hepatic organic cation transporter 1 and hepatic distribution of metformin in high-fat diet-induced obese mice. Drug Metabol. Pharmacokinet..

[269] Chen L., Shu Y., Liang X., Chen E.C., Yee S.W., Zur A.A., Li S., Xu L., Keshari K.R., Lin M.J. (2014). Oct1 is a high-capacity thiamine transporter that regulates hepatic steatosis and is a target of metformin. Proc. Natl. Acad. Sci. USA.

[270] Abdul-Ghani M.A., Muller F.L., Liu Y., Chavez A.O., Balas B., Zuo P., Chang Z., Tripathy D., Jani R., Molina-Carrion M. (2008). Deleterious action of fa metabolites on atp synthesis: Possible link between lipotoxicity, mitochondrial dysfunction, and insulin resistance. Am. J. Physiol. Endocrinol. Metabol..

[271] Zheng H., Yde C.C., Arnberg K., Molgaard C., Michaelsen K.F., Larnkjaer A., Bertram H.C. (2014). Nmr-based metabolomic profiling of overweight adolescents: An elucidation of the effects of inter-/intraindividual differences, gender, and pubertal development. BioMed Research Int..

[272] Gkourogianni A., Kosteria I., Telonis A.G., Margeli A., Mantzou E., Konsta M., Loutradis D., Mastorakos G., Papassotiriou I., Klapa M.I. (2014). Plasma metabolomic profiling suggests early indications for predisposition to latent insulin resistance in children conceived by ICSI. PloS One.

[273] Ekberg N.R., Brismar K., Malmstedt J., Hedblad M.A., Adamson U., Ungerstedt U., Wisniewski N. (2010). Analyte flux at a biomaterial-tissue interface over time: Implications for sensors for type 1 and 2 diabetes mellitus. J. Diabetes Sci. Technol..

[274] Sorriento D., Pascale A.V., Finelli R., Carillo A.L., Annunziata R., Trimarco B., Iaccarino G. (2014). Targeting mitochondria as therapeutic strategy for metabolic disorders. Sci. World J..

[275] Jones L.R., Wilson C.I., Wadden T.A. (2007). Lifestyle modification in the treatment of obesity: An educational challenge and opportunity. Clin. Pharmacol. Therapeut..

